# Cassane diterpenoid ameliorates dextran sulfate sodium-induced experimental colitis by regulating gut microbiota and suppressing tryptophan metabolism

**DOI:** 10.3389/fimmu.2022.1045901

**Published:** 2023-01-19

**Authors:** Ting Liu, Zunxi Ning, Pengyu Liu, Huiyuan Gao

**Affiliations:** ^1^ School of Traditional Chinese Materia Medica, Shenyang Pharmaceutical University, Shenyang, China; ^2^ Key Laboratory of Structure-Based Drug Design & Discovery of Ministry of Education, Shenyang Pharmaceutical University, Shenyang, China

**Keywords:** colitis, caesaldekarin e, anti-inflammation, gut microbiota, metabolomics, tryptophan metabolism

## Abstract

Ulcerative colitis (UC) is one form of inflammatory bowel disease (IBD), characterized by chronic relapsing intestinal inflammation. As increasing morbidity of UC and deficiency of conventional therapies, there is an urgent need for attractive treatment. Cassane diterpenoids, the characteristic chemical constituents of *Caesalpinia* genus plants, have been studied extensively owing to various and prominent biological activities. This study attempted to investigate the bioactivity of caesaldekarin e (CA), a cassane diterpenoid isolated from *C. bonduc* in our previous work, on dextran sulfate sodium (DSS)-induced experimental colitis and clarify the function mechanism. The results indicated that CA ameliorated mice colitis by relieving disease symptoms, suppressing inflammatory infiltration and maintaining intestinal barrier integrity. Furthermore, 16S rRNA gene sequencing analysis indicated that CA could improve the gut microbiota imbalance disrupted by DSS and especially restored abundance of *Lactobacillus*. In addition, untargeted metabolomics analysis suggested that CA regulated metabolism and particularly the tryptophan metabolism by inhibiting the upregulation of indoleamine 2,3-dioxygenase 1 (IDO-1). It also been proved in IFN-γ induced RAW264.7 cells. Overall, this study suggests that CA exhibits anti-UC effect through restoring gut microbiota and regulating tryptophan metabolism and has the potential to be a treatment option for UC.

## 1 Introduction

Inflammatory bowel disease (IBD), characterized by the chronic and relapsing inflammatory disorder of the gastrointestinal tract, generally begin in young adulthood and last throughout life (1). As the two main clinicopathological subtypes of IBD, ulcerative colitis (UC) and Crohn’s disease (CD) show different inflammatory location and histological alterations in the intestinal wall. For the UC, the inflammation is limited to the colon with fewer complications. While the CD involves the entire gastrointestinal tract, usually accompanied by strictures, abscesses, fistulas as well as other complications (2, 3). The etiology of IBD is complex, and interactions between genetic factor, the host immune system and gut microbiota are thought to underlie the development of IBD (2, 4). From birth to death, the human gastrointestinal tract is colonized by a vast and complex community of bacteria that approximately 10-fold of the total number of cells in the human body. Interactions between bacteria and their hosts can be viewed in terms of a continuum between symbiosis, commensalism and pathogenicity, and the relationship between gut microbiota and their hosts can shift from commensalism to pathogenicity in certain disease states (5). The development of novel analysis techniques, such as shut gun sequencing, metagenomics and next-generation sequencing, allow us to bypass the traditional culture-dependent bias and deepen and broaden our understanding of the composition, diversity, and roles of the gut microbiota in human health and diseases (6). IBD is associated with tremendous changes in the composition of gut microbiota, underlining the importance of the microbiota in disease etiology. Notably, dysbiosis and decreased complexity of the gut microbiota have been observed in CD and UC patients (7–9).

The current treatments for IBD are generally divided into two types: nonbiological therapy and biological therapy. Nonbiological therapies such as corticosteroids, immunomodulators and aminosalicylates, characterized by short half-life, low production cost and high patient’s satisfaction from oral administration, have been used for a long time, which can improve clinical symptoms but do not change the overall disease course of IBD (10–12). In addition, these small molecule drugs pose various side effects such as hyperglycemia, hypothalamic-pituitary-adrenal axis suppression, opportunistic infections and osteoporosis for glucocorticoid, leukopenia, increased susceptibility to infection and hepatotoxicity for thiopurines, and headache, nausea and epigastric pain for sulfasalazine (13–15). Biologics are a group of molecules including monoclonal antibodies, recombinant cytokines and specific antagonists of receptors and cytokines that participate in regulating inflammation during immune-mediated process. They are attractive treatment options for patients who poorly or do not respond to small molecule drugs such as steroids or immunosuppressants, or patients suffering from serious adverse reactions of other IBD drugs (16). However, in addition to risks for side effects and patients failing to response, higher manufacturing and quality control costs of biologics impose a burden to economically disadvantaged patients and the healthcare system (17–19). Collectively, the development of safe, effective and economical therapeutics for IBD are urgently needed.

Cassane diterpenoids, the characteristic chemical constituents of medicinal plants of the *Caesalpinia* genus, have attracted considerable interest owing to their significant biological activities including antimalarial, anti-inflammatory, antimicrobial, antitumor, and antioxidant properties (20). Caesalpinin M2, a cassane furanoditerpenoid isolated from the seeds of *C. minax* in our previous work (21), exerted anti-inflammatory effect as a selective glucocorticoid receptor modulator by repressing NF-κB-dependent transcription without inducing glucocorticoid receptor transactivation, providing therapeutic potential in the treatment of inflammatory diseases (22). In this study, caesaldekarin e (CA), a cassane diterpenoid isolated from the seed kernels from *C. bonduc* in our previous work (23), was used to evaluate its anti-inflammatory activity and investigate its effect against DSS-induced experimental colitis.

## 2 Materials and methods

### 2.1 Preparation of chemical and reagents

CA was isolated from seed kernels of *Caesalpinia bonduc* in the laboratory and its purity (>95%) was confirmed by HPLC analysis in our previous study. Its structure was determined by a combination of ^1^H and ^13^C NMR spectra and comparison with reference. Dextran sulfate sodium (DSS, molecular weight 36-50 kDa) was purchased from Meilunbio (Dalian, China). Sulfasalazine (SASP) was purchased from Shanghai Xinyi Tianping Pharmaceutical Co. Ltd. Other chemicals, solvents and reagents were analytical grade.

### 2.2 Cell culture

Murine macrophage RAW264.7 were obtained from the Shanghai Institute of Cell Biology (Shanghai, China) and were cultured in Dulbecco’s modified Eagle’s medium (DMEM) containing 10% fetal bovine serum, penicillin (100 U/mL) and streptomycin (100 μg/mL) in 5% CO_2_ at 37 °C.

### 2.3 Animals

Male BALB/c mice (18-22 g) were purchased from Liaoning Changsheng Biotechnology Co., Ltd (License no. SCXK (Liaoning) 2020-0001). Mice were housed under standard conditions (temperature 23 ± 2 °C and 12 h light/dark cycle) and fed with standard chow pellets and water *ad libitum*. Mice were acclimatized for one week prior to the experiments. The experimental procedure was performed according to the guidelines approved by the Institutional Animal Care and Use Committee of Shenyang Pharmaceutical University.

### 2.4 MTT assay

RAW264.7 cells were cultured in 96-well plates at a density of 1 × 10^4^ overnight, which were subsequently treated with compound CA for 24 h. After removing the medium, MTT was added to the 96-well plate and cultured at 37 °C for 4 h to form formazan. Finally, the formazan was solubilized in DMSO, and the absorbance was detected at 490 nm.

### 2.5 NO generation assay

RAW264.7 cells were seeded in 96-well plates at a density of 2 × 10^4^ and cultured overnight. Cells were pretreated with CA (3.125, 6.25, 12.5, 25 μM) as indicated concentrations for 2 h, then cells were treated with LPS (0.1 μg/mL) for another 24 h. The culture medium was collected to determine the nitrite level using Griess reagent according to the instructions (Beyotime Biotechnology, Shanghai, China).

### 2.6 Induction and treatment of colitis

50 BALB/c mice were randomly divided into five groups: blank control group (Control), DSS-induced UC model group (DSS), CA-treated UC group by intraperitoneal injection (CAip), CA-treated UC group by intragastric administration (CAig) and SASP-treated UC group (SASP). Experimental colitis was induced by administration with distilled water containing 3% DSS (wt/vol) for 7 days, followed by distilled water for the next 2 days. Control group Mice were supplied with distilled water without DSS throughout the experiment. CAig and SASP groups were administered intragastrically with CA (20 mg/kg) or SASP (200 mg/kg) suspended in 0.5% CMC-Na water solution, while the mice in Control, DSS and CAip groups received same volume of 0.5% CMC-Na as vehicle from day 1 to 9. For the CAip group, CA (20 mg/kg, suspended in 0.2 mL saline) was provided daily through intraperitoneal injection for 9 days, and animals in Control, DSS, CAig and SASP groups received 0.2 mL normal saline *via* intraperitoneal injection for 9 days.

### 2.7 Sample collection

The whole blood sample were collected on the 10^th^ day from the retroorbital venous plexus under ether anesthesia. After standing for 1 h at room temperature, the blood was centrifuged at 3500 rpm for 10 min and the serum was transferred to another tube and stored at -80 °C for subsequent metabolites analysis. Next, Mice were sacrificed and the colon was collected to measure the length between the proximal rectum and the ileocecal junction. Then, colon samples were cut into fragments and about 1 cm of distal colon was used for histological analysis. Mouse feces in colon were transferred into sterile centrifuge tubes and stored at -80 °C after liquid nitrogen freezing. The remaining colon tissues were rinsed with normal saline and subsequently stored in a refrigerator at -80 °C for further analysis.

### 2.8 Assessment of disease activity index

The body weight, rectal bleeding and fecal consistency were monitored every day. The disease activity index (DAI) was determined from a combination of following parameters: a) body weight loss (0: none, 1: 1-5%, 2: 5-10%, 3: 10-20%, 4: >20%); b) diarrhea (0: normal, 1: soft but formed, 2: very soft, 3: half diarrhea, 4: diarrhea); c) hematochezia (0: none, 2: slight bleeding, 4: serious bleeding).

### 2.9 Histopathological assessment

The distal colon was cut and fixed in 4% paraformaldehyde, followed by paraffin embedding. Sections (5 μm thick) were stained with hematoxylin and eosin (H&E). The histological damage and inflammation were observed using Eclipse Ci-L microscope (Nikon, Japan).

### 2.10 Measurement of colonic myeloperoxidase activity and cytokines

The MPO activity of colon tissues were measured by a myeloperoxidase assay kit according to the manufacturer’s instructions (Nanjing Jiancheng Biotechnology Company, Nanjing, China). Colon tissues were weighed and homogenized with normal saline on ice and then centrifuged at 3000 rpm for 10 min at 4 °C. The supernatants were collected for the measurements of IL-6, TNF-α and IL-1β using commercial ELISA kit (Servicebio, Wuhan, China). Inflammatory cytokines and markers including IL-6, TNF-α, IL-1β and serum amyloid A (SAA) in serum were also evaluated by ELISA kit (Lianke Biotech Co. Ltd., Hangzhou, China).

### 2.11 Quantitative real-time PCR analysis

Total RNA was extracted from colon tissues using Trizol reagent according to the manufacturer’s instructions (Takara, Japan). Total RNA was reversely transcribed using the PrimeScript RT reagent Kit with gDNA Eraser (Takara, Japan). Real-time PCR was carried out using TB Green Premix Ex Taq II (Takara, Japan) in CFX96 Real-Time PCR Detection System. Relative mRNA levels were calculated using the 2^-ΔΔCt^ method and normalized to β-actin expression. Primer sequences are shown in **Table S1**.

### 2.12 Western blot analysis

Colon tissues of the mice and cultured cells were lysed using RIPA buffer with protease inhibitor and incubated 30 min on ice. The homogenate was centrifuged at 12000 rpm for 10 min at 4 °C and the supernatant was collected. The protein concentration was determined with BCA protein assay kit (Beyotime Biotechnology, China). Total proteins (20 μg) for each sample were separated by SDS-PAGE and subsequently transferred to NC membranes. Membranes were blocked with 5% non-fat milk for 2 h at room temperature, then were incubated overnight at 4 °C with iNOS (1:2000), Occludin (1:2000), Claudin-1 (1:1000) and IDO-1 (1:5000) antibodies (Proteintech Group, Inc., China). Membranes were incubated with horseradish peroxidase (HRP)-conjugated secondary antibody (Proteintech Group, Inc., China) at room temperature for 1 h. Images were detected by chemiluminescence detection system (Amersham Imager 680, Sweden). The obtained chemiluminescence signals were analyzed with Image J software.

### 2.13 Fecal DNA extraction and Illumina Miseq sequencing

Fecal genomic DNA was extracted with kit according to the manufacture’s instruction. Extracted DNA was used as template to amplify the V3-V4 region of bacterial 16S rRNA gene with the primers 341F (CCTAYGGGRBGCASCAG) and 806R (GGACTACNNGGGTATCTAAT). After purifying the PCR products, high-throughput sequencing was performed on Illumina MiSeq PE250 system (Novogene, Beijing, China).

### 2.14 Processing of sequencing data and diversity analysis

The raw 16S rRNA gene sequencing reads were pieced and quality-filtered, followed by removing chimeric sequences to obtain the effective tags for subsequent analysis. Operational taxonomic units (OTUs) were clustered by UPARSE with 97% similarity. Alpha diversity was measured based on the observed OTU number and presented with Chao, Shannon and Simpson indices. Beta diversity was determined by principal coordinate analysis (PCoA) based on the distance matrix. Linear discriminant analysis (LDA) effect size (LEfSe) method was used to identify significantly different biomarkers between groups (LDA score threshold of 4).

### 2.15 UPLC-MS Global profiling of serum metabolites

The refrigerated serum samples were thawed thoroughly before proceeding. 100 μL of each serum sample from Control, DSS, CAip, CAig and SASP groups was mixed with 400 μL of methanol. After vortex mixing, the samples were incubated for 5 min on ice and centrifuged at 15000 g, 4 °C for 20 min. 400 μL of supernatant was transferred into another clean Eppendorf tube and diluted with LC-MS grade water to contain 53% methanol. The samples were subsequently centrifuged at 15000 g, 4 °C for 20 min, and the supernatant was applied to LC-MS/MS analysis.

QC sample was prepared by mixing equal volume of each serum sample. QC samples were analyzed every 10 runs to monitor the instrument and evaluate the stability and reproducibility of LC-MS system throughout the analysis procedure.

The UHPLC-MS/MS analysis was carried out on a Vanquish UHPLC system (Thermo Fisher, Germany) coupled with an Orbitrap Q Exactive™ HF mass spectrometer (Thermo Fisher, Germany). Each sample was injected into a Hypesil Gold column (100 × 2.1 mm, 1.9 μm, Thermo Fisher) with a flow rate of 0.2 mL/min at 40 °C. The mobile phase for the positive polarity mode consisted of eluent A (0.1% formic acid in water) and eluent B (methanol)), and eluent A (5 mM ammonium acetate, pH 9.0) and eluent B (methanol) for the negative polarity mode. The gradient elution program was set as follows: 0-1.5 min, 2% B; 1.5-3.0 min, 2-100% B; 3.0-10.0 min, 100% B; 10.0-10.1 min, 100-2% B; 10.1-12.0 min, 2% B. For mass spectrometry analysis, the Q Exactive HF mass spectrometer with an electrospray ionization source was used. The parameters employed were as follows: ESI positive and negative mode; mass range, m/z 100-1500 Da; spray voltage, 3.5 kV; capillary temperature, 320 °C; sheath gas flow rate, 35 psi; aux gas flow rate, 10 L/min; S-lens RF level, 60; aux gas heater temperature, 350 °C.

The raw data files obtained from UHPLC-MS/MS were imported into Compound Discoverer 3.1 to perform peak alignment, peak picking and quantitation for each metabolite. The main parameters were as follows: retention time tolerance, 0.2 min; actual mass tolerance, 5 ppm; signal intensity tolerance, 30%; signal/noise ratio, 3, et al. After that, peak intensities were normalized to the total spectral intensity. The normalized date was used to predict the molecular formula based on additive ions, molecular ion peaks and fragment ions. Finally, the peaks were matched with mzCloud, mzVault and MassList database to obtain the accurate qualitative and relative quantitative results.

KEGG database (https://www.genome.jp/kegg/), HMDB database (https://hmdb.ca/metabolites) and LIPID MAPS database (http://www.lipidmaps.org/) were used to annotate identified metabolites. Partial least squares discriminant analysis (PLS-DA) was performed at metaX. Statistical significance (P-value) was calculated by univariate analysis (t-test). The metabolites with variable importance in projection (VIP) > 1, P-value < 0.05, and fold change (FC) > 1.2 or FC < 0.83 were considered as differential metabolites.

### 2.16 Statistical analysis

All data were expressed as mean ± standard error of the mean (SEM). Statistical analysis was conducted by one-way analysis of variance (ANOVA), followed by Tukey’s test using GraphPad Prism 9.0.0. ^#^ *P* < 0.05, ^##^ *P* < 0.01, ^###^ *P* < 0.001 *vs.* Control group, ^*^ *P* < 0.05, ^**^ *P* < 0.01, ^***^ *P* < 0.001 *vs.* DSS group.

## 3 Results

### 3.1 CA exhibited anti-inflammatory effect in macrophages

**Figure 1:**
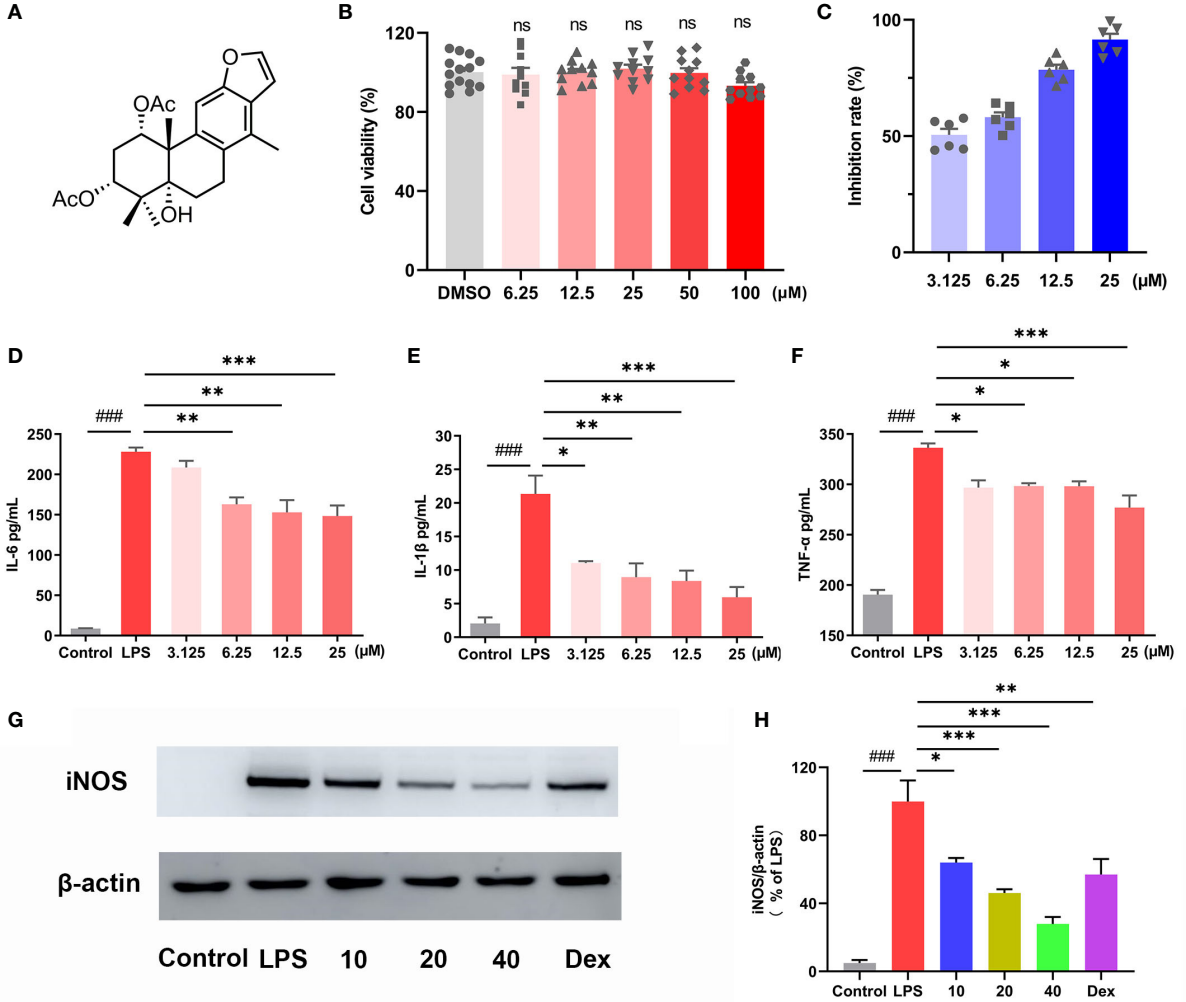
CA exhibited anti-inflammatory effect in macrophages. **(A)** Chemical structure of caesaldekarin e (CA). **(B)** RAW264.7 cells were treated with CA for 24 h as indicated concentrations. The cell viability was determined by MTT assay. **(C)** RAW264.7 cells were pretreated with the indicated concentration of CA for 2 h, followed by LPS (0.1 μg/mL) stimulation for another 24 (h) Then, the culture medium was collected to determine the nitrite levels using the Griess reagent. RAW264.7 cells were pretreated with the indicated concentration of CA for 2 h, followed by LPS (0.1 μg/mL) stimulation for another 24 (h) The culture medium was collected to determine the levels of IL-6 **(D)**, IL-1β **(E)** and TNF-α **(F)** by ELISA. **(G)** RAW264.7 cells were pretreated with CA (10, 20, 40 μM) for 2 h and stimulated with LPS (0.1 μg/mL) for another 12 (h) Then, iNOS protein expression was determined by Western blot analysis. **(H)** The relative protein expression of iNOS was normalized to β-actin. Data are expressed as the mean ± SEM of three independent experiments. ^###^ *P* < 0.001 *vs.* Control group, ^*^ *P* < 0.05, ^**^ *P* < 0.01, ^***^ *P* < 0.001 *vs.* LPS group. ns, no significant difference with DMSO treatment.

MTT assay was used to test the cytotoxicity of CA (**Figure 1A**), and the results indicated that CA did not affect the cell viability even at the dosage of 100 μM (**Figure 1B**). Then, LPS-stimulated RAW264.7 cells were used to investigate the anti-inflammatory activity of CA. Nitric oxide (NO) is a free radical acting as a cellular signaling molecule, mainly produced by inducible nitric oxide synthase (iNOS), which has been associated with the pathophysiological of inflammation (24, 25). In the present study, LPS stimulation caused dramatic increase of NO in RAW264.7 cells, however, CA treatment significantly inhibited the production of NO in a dose-dependent manner (**Figure 1C**). ELISA assay revealed that CA dose-dependently inhibited the release of inflammatory cytokines including IL-6, IL-1β and TNF-α induced by LPS (**Figures 1D, E, F** **)**. Furthermore, CA concentration-dependently inhibited the overexpression of iNOS protein induced by LPS (**Figures 1G–H** **)**. Overall, CA effectively suppressed inflammatory response in RAW264.7 cells.

### 3.2 CA administration ameliorated the clinical symptoms of DSS-induced colitis

**Figure 2:**
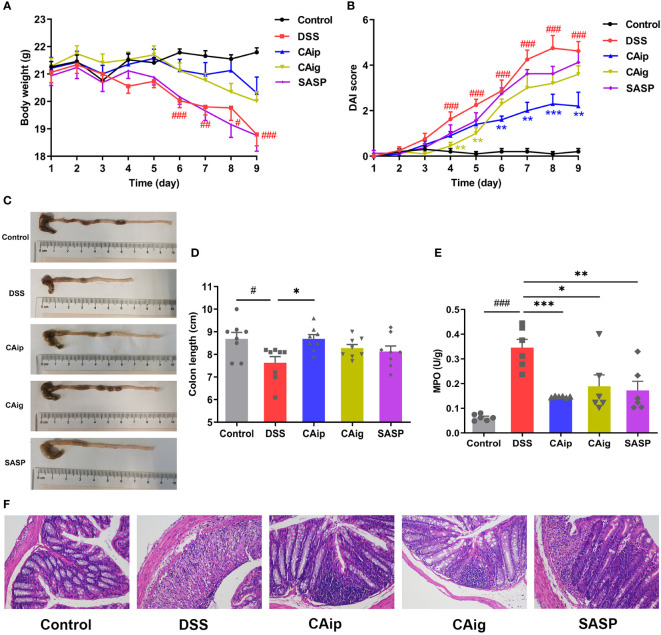
CA treatment ameliorated DSS-induced experimental colitis. **(A)** Body weight change. **(B)** Disease activity index (DAI) score. **(C)** Representative pictures of colon appearance and colon length. **(D)** colon length. **(E)** MPO activity. **(F)** Representative microscopic pictures of H&E staining (200 × magnification). **(A, B, D)** Data are presented as the mean ± SEM (n = 8~10). **(E)** Data are presented as the mean ± SEM (n = 6). ^#^ *P* < 0.05, ^##^ *P* < 0.01, ^###^ *P* < 0.001 *vs.* Control group, ^*^ *P* < 0.05, ^**^ *P* < 0.01, ^***^ *P* < 0.001 *vs.* DSS group.

A UC mouse model was induced through 3% DSS administration in drinking water for 7 days. In the course of experiment, DSS group showed body weight loss, diarrhea and hematochezia. By contrast, treatment with CA (CAip and CAig) and SASP alleviated these pathological alterations and decreased DAI scores (**Figures 2A, B** **)**. The colon length of DSS group was evidently shorter than that of the control group, while DSS-induced colon shortening was improved by treatment with CA and SASP, especially by CA *via* intraperitoneal injection (**Figures 2C, D** **)**.

### 3.3 CA suppressed inflammatory infiltration of colon tissue

Histopathological evaluations of colon were conducted by H&E staining and representative results were shown in **Figure 2F**. In the control group, the colon tissues showed intact mucosa, submucosa, muscular layer and outer membrane. However, the colon from DSS group showed disruption of the epithelial layer, loss of goblet cells, crypts loss and inflammatory cell infiltration. However, CA and SASP treatment had less distortion of epithelium, relative integral crypt structures and fewer infiltration of inflammatory cells.

Neutrophil infiltration leads to remarkable elevation of MPO activity in colon, which is a typical inflammatory marker of colitis (26). As shown in **Figure 2E**, MPO activity of DSS group significantly higher than that of the control group, while CA and SASP treatment significantly suppressed the elevation of MPO activity. Compared with DSS group, the reductions in CAip, CAig and SASP groups were 70.8%, 55.4% and 61.2%, respectively. Taken together, these results suggested that CA exhibited therapeutic effect on DSS-induced colitis through inhibiting inflammatory infiltration.

### 3.4 CA decreased the level of inflammatory markers in colon tissue and serum

**Figure 3:**
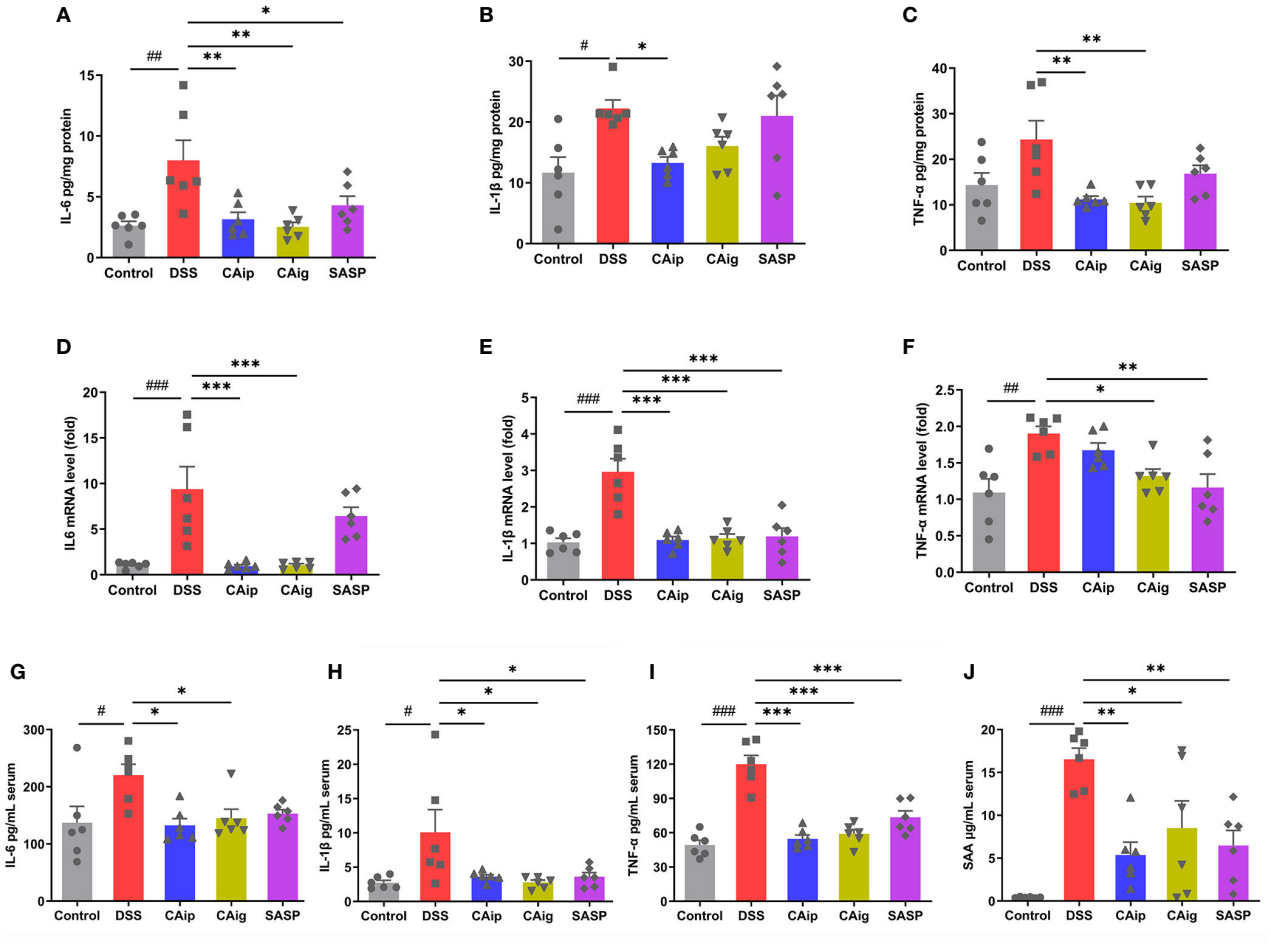
Effects of CA on inflammatory cytokines IL-6 **(A)**, IL-1β **(B)** and TNF-α **(C)** by ELISA and mRNA expression of IL-6 **(D)**, IL-1β **(E)** and TNF-α **(F)** by qRT-PCR in colon tissue. Effects of CA on inflammatory markers IL-6 **(G)**, IL-1β **(H)**, TNF-α **(I)** and SAA **(J)** in serum by ELISA. Data are expressed as the mean ± SEM (n = 6). ^#^ *P* < 0.05, ^##^ *P* < 0.01, ^###^ *P* < 0.001 *vs.* Control group, ^*^ *P* < 0.05, ^**^ *P* < 0.01, ^***^ *P* < 0.001 *vs.* DSS group.

To investigate the effect of CA on the release of pro-inflammatory cytokines in the colon, colon tissues were collected and the expression of pro-inflammatory cytokines were measured by ELISA and qRT-PCR. As shown in **Figure 3**, the levels of pro-inflammatory cytokines including IL-6, IL-1β and TNF-α in colon tissue significantly increased in the DSS group. However, the administration of CA markedly reduced the levels of these cytokines. Notably, the effects of CA for decreasing the level of pro-inflammatory cytokines were superior to SASP. Similarly, DSS caused considerable increase of inflammatory markers including IL-6, IL-1β, TNF-α and SAA in serum, which also been reduced through CA treatment.

### 3.5 CA improved intestinal barrier by enhancing TJ protein expression

**Figure 4:**
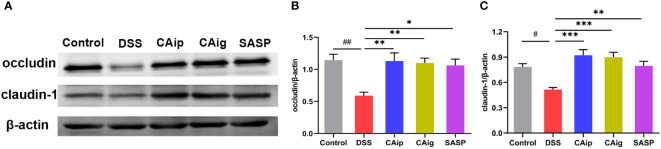
CA protected intestinal epithelial barrier by enhancing TJs proteins. Representative western blotting images of occludin and claudin-1 **(A)**, and the relative protein expressions were normalized to β-actin **(B, C)**. Data are shown as the mean ± SEM (n = 5). ^#^ *P* < 0.05, ^##^ *P* < 0.01 *vs.* Control group, ^*^ *P* < 0.05, ^**^ *P* < 0.01, ^***^ *P* < 0.001 *vs.* DSS group.

Intestinal barrier integrity is a prerequisite for mucosal functional homeostasis and one of the main causes of several gastrointestinal diseases such as IBD (27). In addition to depending on coordinated proliferation and cell death, barrier function is also determined by tight junction (TJ), the paracellular barrier of intestinal epithelium. The TJ proteins responsible for barrier and passage function consist of 2 families: the TAMP family consisting of occludin, tricellulin and marvelD3, and claudins family (28). To investigate the effect of CA on intestinal barrier function, the expression levels of TJ proteins occludin and claudin-1 were detected by western blot. As shown in **Figure 4**, compared with control group, occludin and claudin-1 were obviously down-regulated in DSS group, indicating that the TJ structure was disrupted. By contrast, CA treatment markedly enhanced occludin and claudin-1 expression, suggesting that CA could improve intestinal integrity.

### 3.6 CA altered gut microbiota diversity and composition

**Figure 5:**
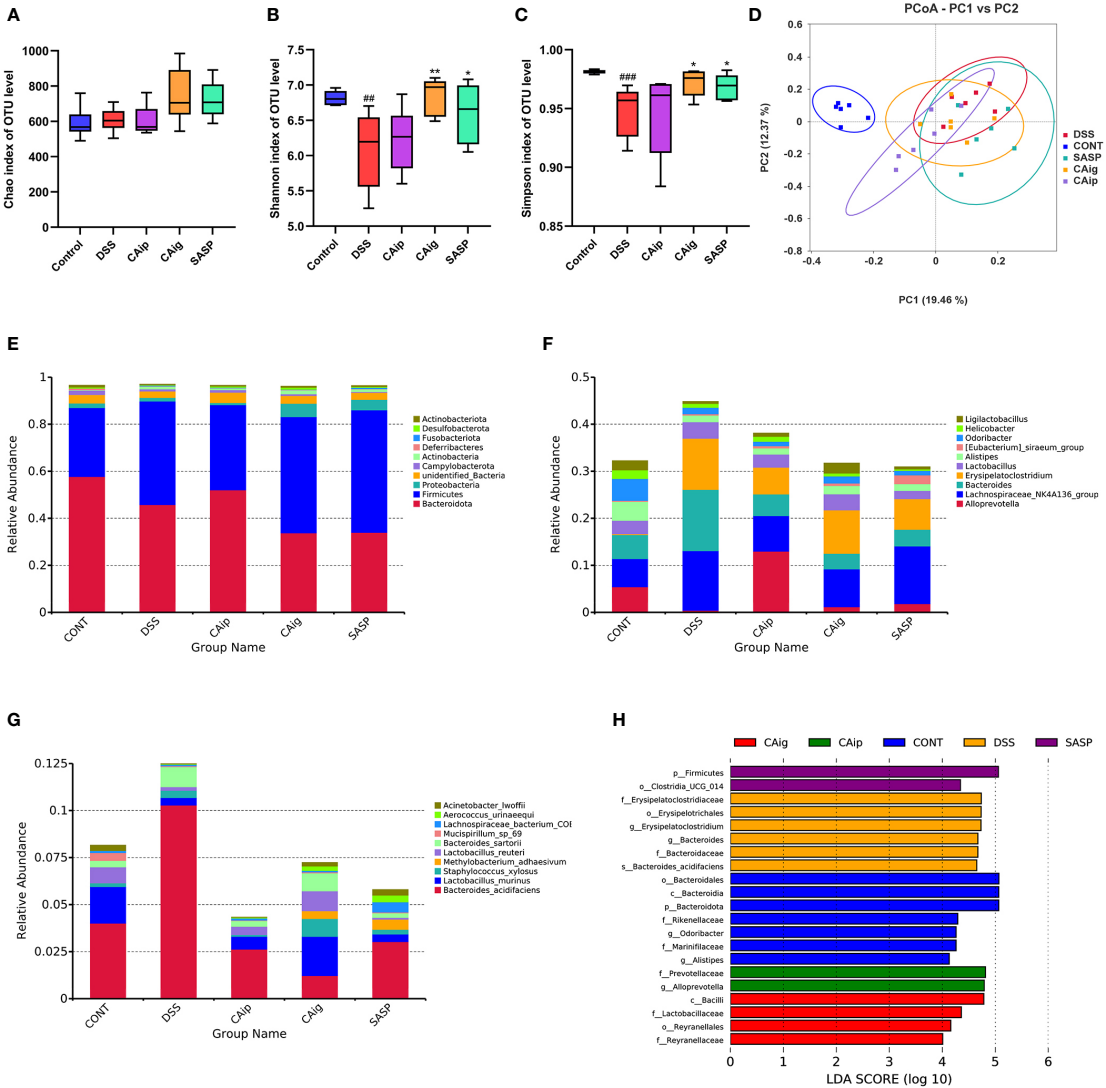
CA treatment altered the gut microbiota diversity and composition. Alpha diversity estimated by the Chao **(A)**, Shannon **(B)** and Simpson **(C)** indices of OTU level. **(D)** Principal coordinate analysis (PCoA) using Bray-Curtis metric distances of beta diversity. The relative abundance of fecal microbiota in the top 10 of phylum **(E)**, genus **(F)** and species **(G)** levels. **(H)** The LEfSe analysis. The criterion is log LDA score > 4.0. n = 6 for each group. ^##^ *P* < 0.01, ^###^ *P* < 0.001 vs. Control group, * *P* < 0.05, ** *P* < 0.01 vs. DSS group.

To determine whether CA treatment changed the microbiome, 16S rRNA sequencing was performed for fecal samples of each group. We next compared the alpha diversity and beta diversity among different groups. The alpha diversity of a sample reflects the richness and diversity of the microbial community. As shown in **Figures 5A–C**, compared with the control group, DSS-treated colitis group exhibited extremely significant reduction of diversity (Shannon and Simpson), whereas there was no significant difference of microbial species richness (Chao) among the five groups. Notably, CA oral administration and SASP treatment markedly improved the microbial diversity.

The Beta diversity was displayed in **Figure 5D**. The principal coordinate analysis (PCoA) based on Bray-Curtis distance at OUT level showed overall structure shift of gut microbiota in mice after DSS challenge compared with that of the normal mice. Although CA and SASP treatment could not completely reverse the change of gut microbiota, they still partially mitigate the abnormal gut microbiota in DSS-induced UC mice.

### 3.7 Gut microbiota composition of UC mice at different level

Histograms were used to illustrate the microbial community structure and show the differences in the relative abundance of major microbiota at different levels. In terms of bacterial composition at the phylum level, all groups possessed similar taxonomic communities mainly composed of Firmicutes, Bacteroidetes, Proteobacteria, Actinobacteria, Fusobacteria and Desulfobacteria. Compared with control group, Firmicutes and Fusobacteria were enriched, while Desulfobacteria and Actinobacteria were reduced in DSS group. However, CAip and CAig treatment could increase the level of Desulfobacteria, and CAig and SASP could increase the level of Actinobacteria (**Figure 5E**, **Supplementary Figure 1**). Taxonomic compositions of each group were also compared at the class, order and family levels (**Supplementary Figures 2–5**). At the genus level, bacterial genera that ranked top ten in relative abundance were analyzed. As shown in **Figure 5F** and **Supplementary Figure 6**, the DSS-treated group exhibited significantly increased proportions of *Bacteroides* and *Erysipelatoclostridium*, but decreased proportions of *Alloprevotella*, *Alistipes*, *Odoribacter* and *Ligilactobacillus* compared to the control group. However, CAip and CAig treatment could reduce the proportion of *Bacteroides*, and notably, CAig could increase the abundance of *Ligilactobacillus*. Besides, compared with the control group, the relative abundances of *Lactobacillus murinus* and *Lactobacillus reuteri* were significantly decreased, whereas *Bacteroides sartorii* was obviously increased in the DSS-induced colitis group. However, the disorder of the microbiota community could be partially restored by CAip and CAig treatment (**Figure 5G**, **Supplementary Figure 7**).

Linear discriminant analysis effect size (LEFSe) analysis was applied to identification of significant biomarkers and dominant bacterial community that might be responsible for the impact on DSS-induced colitis mice in each group. As shown in **Figure 5H**, the genus *Erysipelatoclostridium* (the order Erysipelotrichales and the family Erysipelatoclostridiaceae) and *Bacteroides acidifaciens* (the family Bacteroidaceae and the genus *Bacteroides*) were the crucial bacteria leading to gut microbiota dysbiosis in the DSS group. Nevertheless, Prevotellaceae (the family and the genus *Alloprevotella*) were identified to be the predominant microbiota in CAip group, which might be correlated with its improvement on DSS-induced colitis. Furthermore, Lactobacillaceae (the family and the class Bacilli) and Reyranellaceae (the family and the order Reyranellales) relatively enriched in CAig group, which might be associated with its ameliorating effect on colitis. Taken together, CA treatment, oral administration in particular, significantly altered the gut microbiota diversity and composition.

### 3.8 Identification of metabolite biomarkers through metabolomics

**Figure 6:**
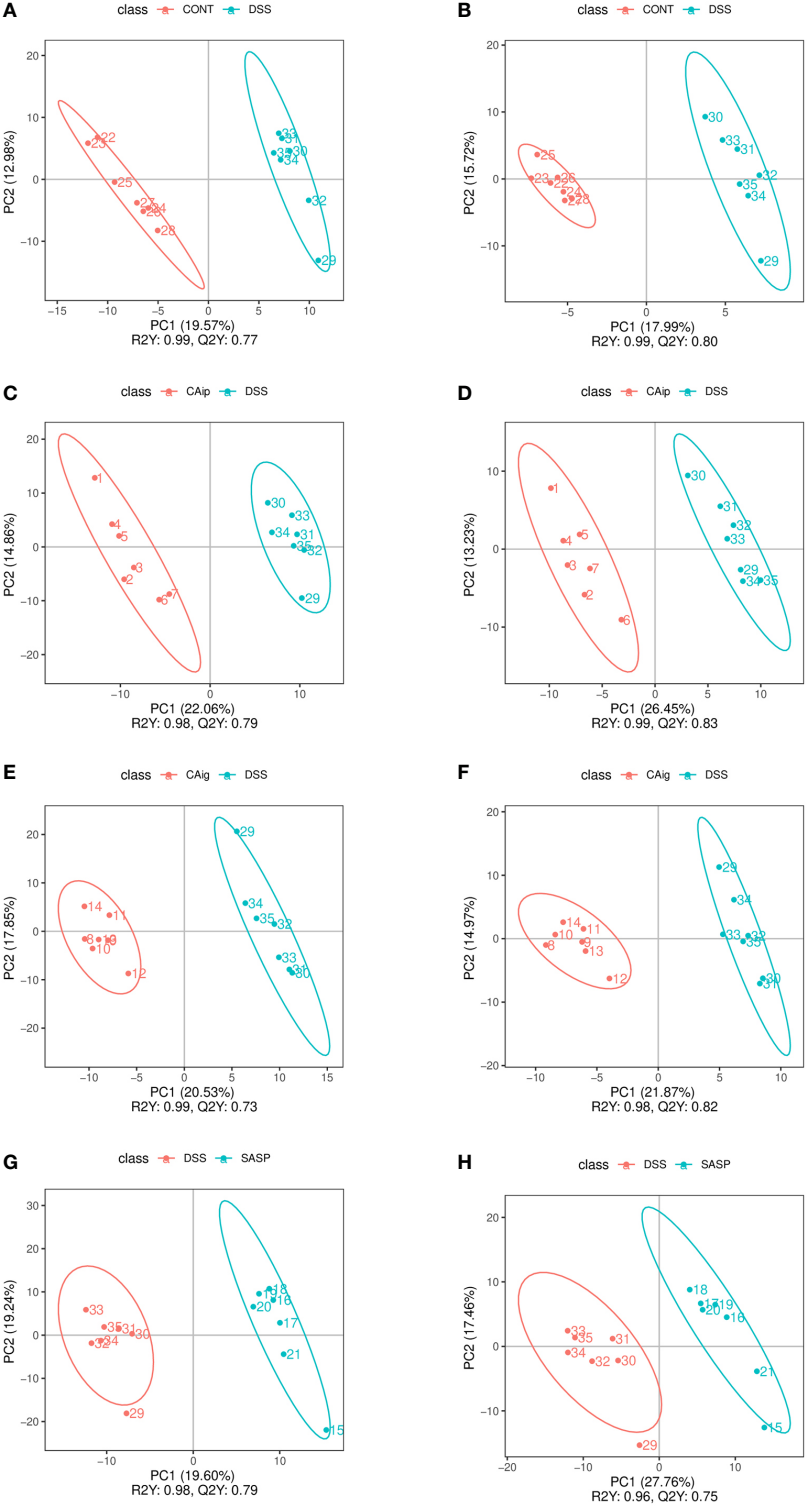
PLS-DA analysis of Control *vs.* DSS **(A, B)**, DSS *vs.* CAip **(C, D)**, DSS *vs.* CAig **(E, F)**, and DSS *vs.* SASP **(G, H)** groups in positive ion and negative ion modes.

Partial least squares discrimination analysis (PLS-DA) was employed to evaluate metabolic variations between groups based on the metabolomics data obtained from both ESI+ and ESI- modes. As shown in **Figure 6**, metabolic phenotype separations were observed between Control *vs.* DSS, DSS *vs.* CAip, DSS *vs.* CAig, and DSS *vs.* SASP groups, indicating that there were remarkable variations in endogenous metabolites among five groups. As shown in **Supplementary Table 2**, 80 metabolites in serum were changed obviously in the DSS group compared to those of control group, whereas 30, 31 and 17 metabolites were reversed by CAip, CAig and SASP treatment, respectively. In addition, a considerable number of metabolites showed no significant difference between the Control and DSS groups, but changed obviously after CA or SASP treatment.

Through the KEGG pathway enrichment analysis of serum differential metabolites, potential metabolic pathways were identified to separate the above groups. As shown in **Supplementary Figures 8, 9**, metabolic pathways that were perturbed in DSS group mainly including neuroactive ligand-receptor interaction, synaptic vesicle cycle, gastric acid secretion, insulin resistance, fatty acid degradation, glutathione metabolism, and tryptophan metabolism. Tryptophan metabolism, phenylalanine metabolism, phenylalanine, tyrosine and tryptophan biosynthesis, aldosterone synthesis and secretion, pyruvate metabolism, and protein digestion and absorption, participated in the therapeutic effect of CA *via* intraperitoneal injection. Tryptophan metabolism, phenylalanine metabolism, pyruvate metabolism, protein digestion and absorption, oxidative phosphorylation, and nicotinate and nicotinamide metabolism were the key metabolic pathways in the CA treatment through oral administration. SASP-mediated metabolic pathways were focused on the glutathione metabolism, nicotinate and nicotinamide metabolism, arachidonic acid metabolism, vitamin digestion and absorption, regulation of lipolysis in adipocytes, and tryptophan metabolism. Collectively, tryptophan metabolism was the common pathway of CA and SASP regulating the metabolites of colitis mice.

### 3.9 CA regulated tryptophan metabolism *via* inhibiting IDO-1

**Figure 7:**
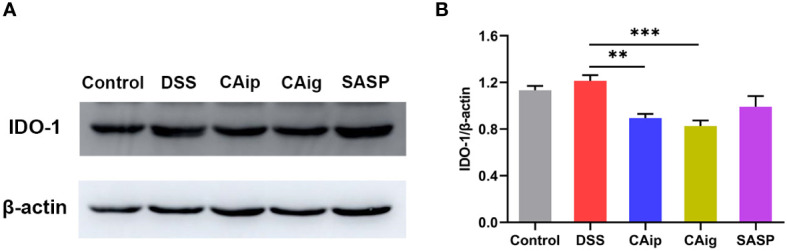
Effect of CA on the protein expression of IDO-1 in colon tissue. Representative western blotting image **(A)**, and the relative protein expression was normalized to β-actin **(B)**. Data are shown as the mean ± SEM (n = 5). ^**^ *P* < 0.01, ^***^ *P* < 0.001 *vs.* DSS group.

Tryptophan is an essential amino acid and is metabolized through three major pathways in the intestines: kynurenine pathway in the immune cells and intestinal lining, serotonin pathway in the enterochromaffin cells, and indole pathway in the gut microbiota (29). About 90-95% of dietary tryptophan is metabolized through kynurenine pathway mediated by the rate-limiting enzyme indoleamine 2,3-dioxygenase 1 (IDO-1) in the gut, leading to the production of kynurenine and downstream products such as kynurenic acid, 3-hydroxykynurenine, 3-hydroxyanthranilic acid, xanthurenic acid and quinolinic acid (30). The metabolomics analysis demonstrated that the levels of kynurenine and kynurenic acid in serum significantly increased in DSS group. However, treatment with CA both *via* oral administration and intraperitoneal injection, dramatically reduced the levels of kynurenine and kynurenic acid, and even decreased the levels of other downstream metabolites including N-formylkynurenine, xanthurenic acid and 3-hydroxyanthranilic acid (**Supplementary Table 2**). We thus investigated whether DSS increased the expression of IDO-1 in colon tissue and CA regulated its expression. The result showed that DSS could induce the upregulation of IDO-1 to some degree but no statistical difference. However, the expression of IDO-1 was significantly reduced by CAip and CAig treatment (**Figure 7**).

### 3.10 CA inhibited IDO-1 expression in IFN-γ induced RAW264.7 cell

**Figure 8:**
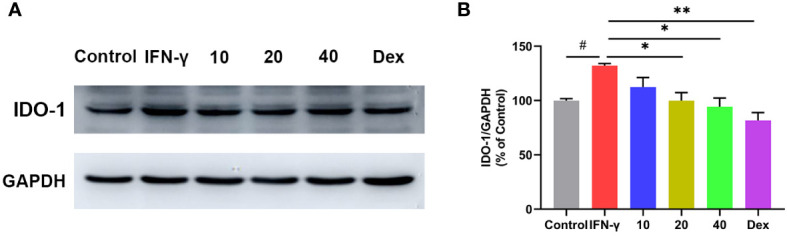
Effect of CA on the protein expression of IDO-1 in INF-γ induced RAW264.7 cells. Representative western blotting image **(A)**, and the relative protein expression was normalized to GAPDH **(B)**. Data are shown as the mean ± SEM of three independent experiments. ^#^ *P* < 0.05 *vs.* Control group, ^*^ *P* < 0.05, ^**^ *P* < 0.01 *vs.* INF-γ group.

IDO-1 is expressed in a variety of tissues and cell types, either constitutively or in response to stimulation associated with inflammatory and immune stimuli. Interferon gamma (IFN-γ) is considered to be the most effective IDO-1 inducer in a range of cell types including dendritic cells and macrophages (31, 32). To further confirm the effect of CA on IDO-1 expression, RAW264.7 cells were pretreated with CA for 2 h, followed by IFN-γ (0.2 μg/mL) incubation for 8 h. Western blot analysis showed that IFN-γ stimulation remarkedly increased the expression of IDO-1 in RAW264.7, however, CA treatment significantly inhibited the upregulation of IDO-1 (**Figure 8**).

## 4 Discussion

**Figure 9:**
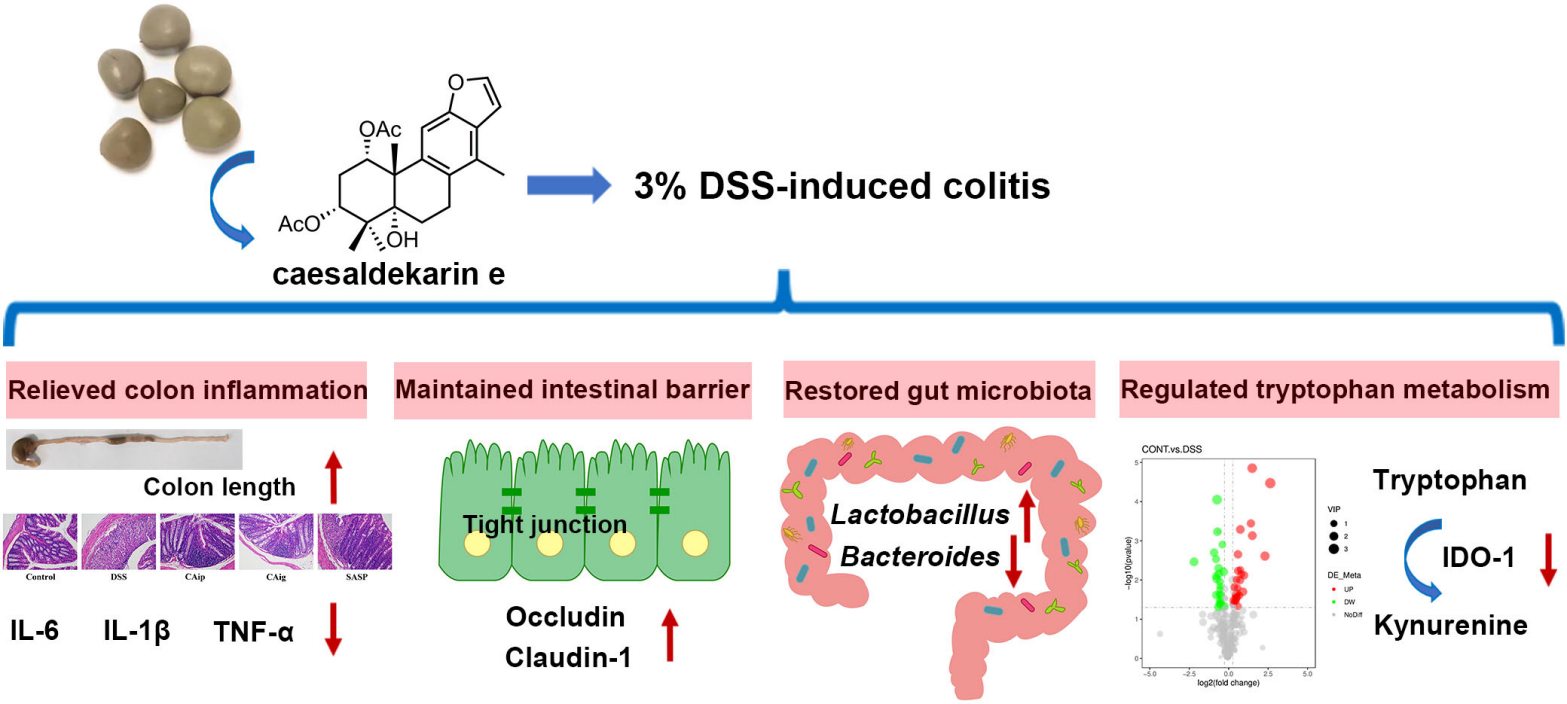
Graphical abstract of this article. The up arrow (↑) and down arrow (↓) represent upregulation and downregulation effects of CA, respectively.

In the present study, we found that treatment with CA both in oral administration and intraperitoneal injection alleviated DSS-induced experimental colitis in mice. Administration with CA maintained the body weight, decreased DAI score, improved colon length and ameliorated inflammatory cell infiltration. The therapeutic effects of CA were attributed to relieving inflammation, improving intestinal barrier, restoring the disrupted gut microbiota, and modulating the metabolites (**Figure 9**). These results provided insights into the protective effects of CA on colon inflammation.

Organisms used as probiotics are most frequently of the lactic acid bacteria and *Bifidobacterium* species and are included in many functional foods and dietary supplements. The main mechanisms of action of probiotics include: 1) colonization and modulation of disordered intestinal microbial communities in children and adult; 2) competitive exclusion of pathogens and bacteriocin production; 3) enzymatic activities and production of volatile fatty acids; 4) cell adhesion and mucin production; 5) modulation of the immune system; and 6) interaction with the brain-gut axis (33, 34). *Lactobacillus* are a major component of symbiotic microbiota of mammals and are one of the most commonly used probiotics (35). Studies suggested that a variety of diseases, including infectious disease, irritable bowel disease, IBD, rheumatoid arthritis, obesity, multiple sclerosis, type 1 diabetes, type 2 diabetes, cancer, and cognitive development and behavior, are correlated with the notable variation of *Lactobacillus* in intestinal abundance. Moreover, probiotic *Lactobacillus* administration play a broader role in the prevention and mitigation of part of aforementioned diseases (36). *L. reuteri*, one species of *Lactobacillus*, possessing features of surviving in low pH and enzyme-rich environment, adhering to epithelium for host-probiotic interaction, competition with pathogenic microorganisms and safety, endows it with great probiotic properties (37). Numerous studies have demonstrated that *L. reuteri* can mitigate experimental colitis by maintaining intestinal immune homeostasis through stimulating dendritic cell maturation and IL-10 production (38, 39), modulating gut microbiota and metabolic disorders (40), increasing mucus thickness and tightening epithelium (41), and decreasing bacterial translocation from mucosa to mesenteric lymph nodes (42, 43). In the present study, we observed that DSS treatment caused significantly reduced abundance of *L. reuteri* compared with the control group. However, the decline was reversed by CA oral administration, suggesting that the increase of *L. reuteri* contributed to the therapeutic effect of CA against DSS-induced colitis. Previous research has shown that *L. murinus* could induce Treg cell expansion, thereby providing resistance against experimental colitis (44). Interestingly, in our current study, the abundance of *L. murinus* disrupted by DSS was also restored through CAig treatment. To further identify the potential biomarkers and dominant bacteria regulated by CA treatment, LEfSe analysis was performed in each group. The family Prevotellaceae and the genus *Alloprevotella* were relatively enriched in CAip group. Consistent with our results, the genus *Alloprevotella* was demonstrated to be lower in DSS-induced experimental colitis, which was generally considered to be short chain fatty acid producer and its abundance was inversely correlated with inflammation (45, 46). Collectively, the alleviative effects of CA on the experimental colitis might be attributed to its gut microbiota modulation activity.

Metabolomics is a technique with advantages of high throughput, satisfactory sensitivity and accuracy for the qualitative and quantitative analysis of small molecule metabolites from biological samples including serum, plasma, urine, feces, breath and biopsy samples, which can not only achieve the quantification of change in a single metabolite but also integrate the changes of multiple metabolites through multivariate analysis to gain a holistic comprehension of metabolic profile (47). In view of heterogeneity, complex etiology and easy recurrence of IBD, metabolomics has bounced into IBD in recent years for assessing disease activity, elucidating the underlying mechanisms, predicting the therapeutic response, and monitoring the disease relapse (47, 48). In this study, untargeted metabolomics analysis of serum was conducted in each group to clarify the mechanism of CA on experimental colitis. As expected, we observed that DSS treatment caused remarkable changes of metabolomic profile covering fatty acids, glycerophospholipids, sphingolipids, sterol lipids, eicosanoids, and amino acids, which could be partially reversed by CA or SASP administration. Through the KEGG pathway enrichment analysis for differential metabolites, tryptophan metabolism was considered to be the important pathway affected by colitis and closely associated with the therapeutic effects of CAip and CAig. Consistently, other studies also reported disturbed metabolites associated with tryptophan metabolism in DSS-induced experimental colitis (49, 50). Similar to our findings, studies on IBD patients have reported that, compared with normal subjects, serum concentration of kynurenine and kynurenic acid significantly increased and IDO was overexpressed in IBD patients, and IDO expression was positively associated with disease activity (51, 52). Treatment with CAip and CAig reduced the level of kynurenine, kynurenic acid and other downstream metabolites, and even downregulated the expression of IDO-1 in colitis mice. Furthermore, CA also inhibited the expression of IDO-1 in IFN-γ induced RAW264.7 cells.

Lipids have several major functions in the organism, including as structural components of cell membranes, energy storage, signal molecules, protein recruitment platforms and substrates for protein-lipid modification (53). Numerous studies have shown that there existed significant disorder of lipid metabolism in IBD patients and animal models (54–56). Phosphatidylcholines (PC), the most abundant phospholipid in all mammalian cell types and subcellular organelles, can be digested by phospholipase A2 to produce lysophosphatidylcholine (LPC) (57). The decrease of PC content and increase of LPC/PC ratio were observed in UC patients (56, 58). Consistently, the present study demonstrated that the PC significantly reduced and LPC content increased in DSS group, with respect to controls. However, the PC concentrations were restored by CA treatment especially *via* intraperitoneal injection. Besides, the contents of sphingomyelin (SM) and lysophosphatidylethanolamine (LPE) declined in the DSS group, and similar results were obtained from previous studies on patients with IBD (56, 59, 60). Treatment with CA or SASP increased the levels of SM and LPE downregulated by DSS, and even promoted those showing no difference between the Control and DSS groups. Overall, CA could effectively regulate the metabolic disorder of phospholipid caused by experimental colitis.

In conclusion, current results demonstrated that CA effectively ameliorated DSS-induced experimental colitis. CA could obviously relieve disease symptoms, suppress inflammatory infiltration and restore intestinal barrier integrity. Meanwhile, CA regulated the disturbance of gut microbiota, particularly by increasing the abundance of *Lactobacillus* and decreasing the abundance of *Bacteroides*. Furthermore, the therapeutic effects of CA might be associated with its modulation on tryptophan metabolism, evidenced by CA inhibited the upregulation of IDO-1 in colon tissue of colitis mice and IFN-γ induced RAW264.7 cells.

## Data availability statement

The datasets presented in this study can be found in the NCBI Sequence Read Archive (SRA) repository under the project number PRJNA880572.

## Ethics statement

The animal study was reviewed and approved by the Institutional Animal Care and Use Committee of Shenyang Pharmaceutical University.

## Author contributions

HG and TL conceived and designed the experiments. TL, ZN and PL performed the experiments. TL analyzed the data and wrote the paper. All authors have read and approved the final manuscript.
