# Supplementary material for: Cassane diterpenoid ameliorates dextran sulfate sodium-induced experimental colitis by regulating gut microbiota and suppressing tryptophan metabolism
Source: Front Immunol. 2023 Jan 19;13:1045901. doi: 10.3389/fimmu.2022.1045901 (PMC9893013; doi:10.3389/fimmu.2022.1045901)
Supplement: Supplementary file 1 [file DataSheet_1.docx]

Supplementary Material

# Supplementary Figures and Tables

## Supplementary Figures

**
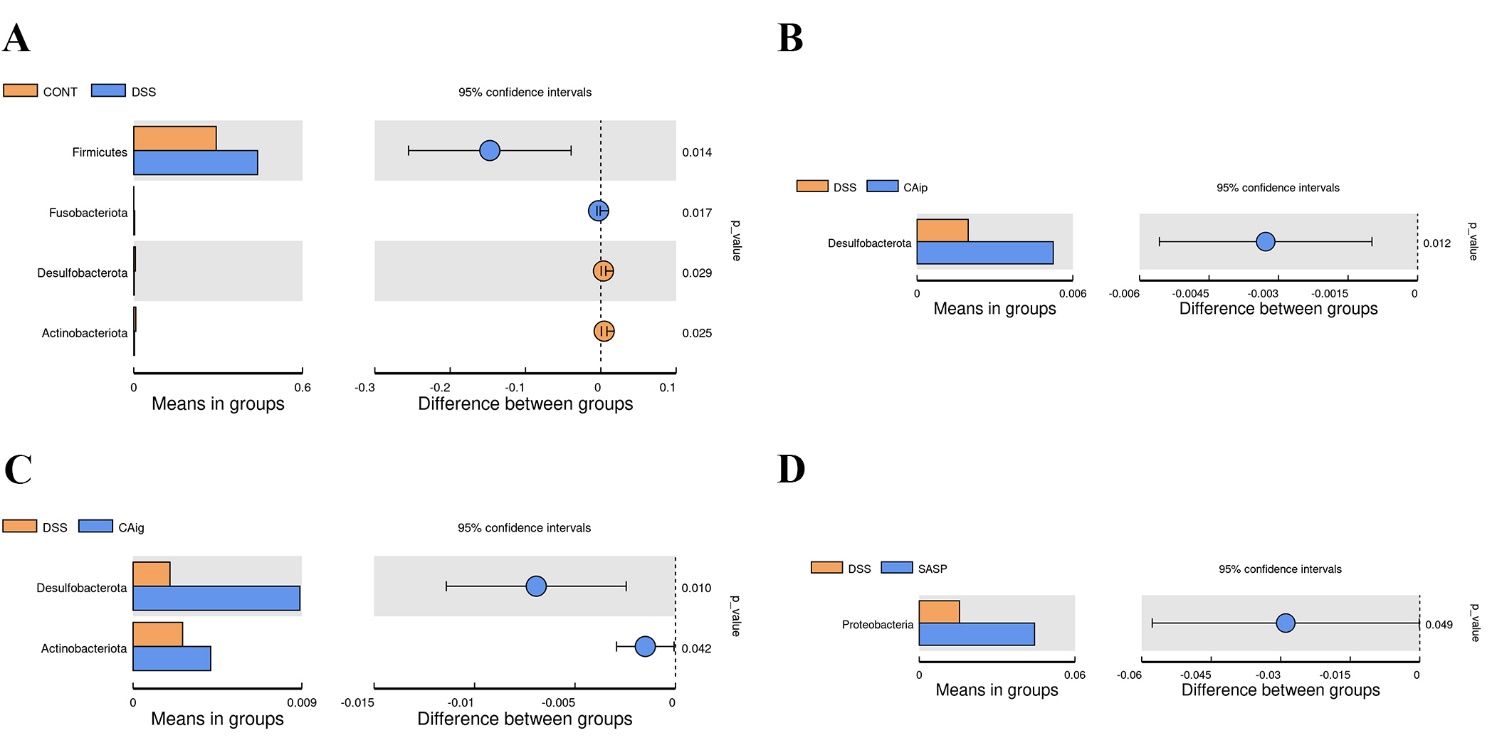
**

**Supplementary Figure 1.** Analysis of different species between groups at phylum level. (**A**) Control *vs* DSS; (**B**) DSS *vs* CAip; (**C**) DSS *vs* CAig; (**D**) DSS *vs* SASP. Statistical significance was evaluated by the Student’s T-test, *P* < 0.05.


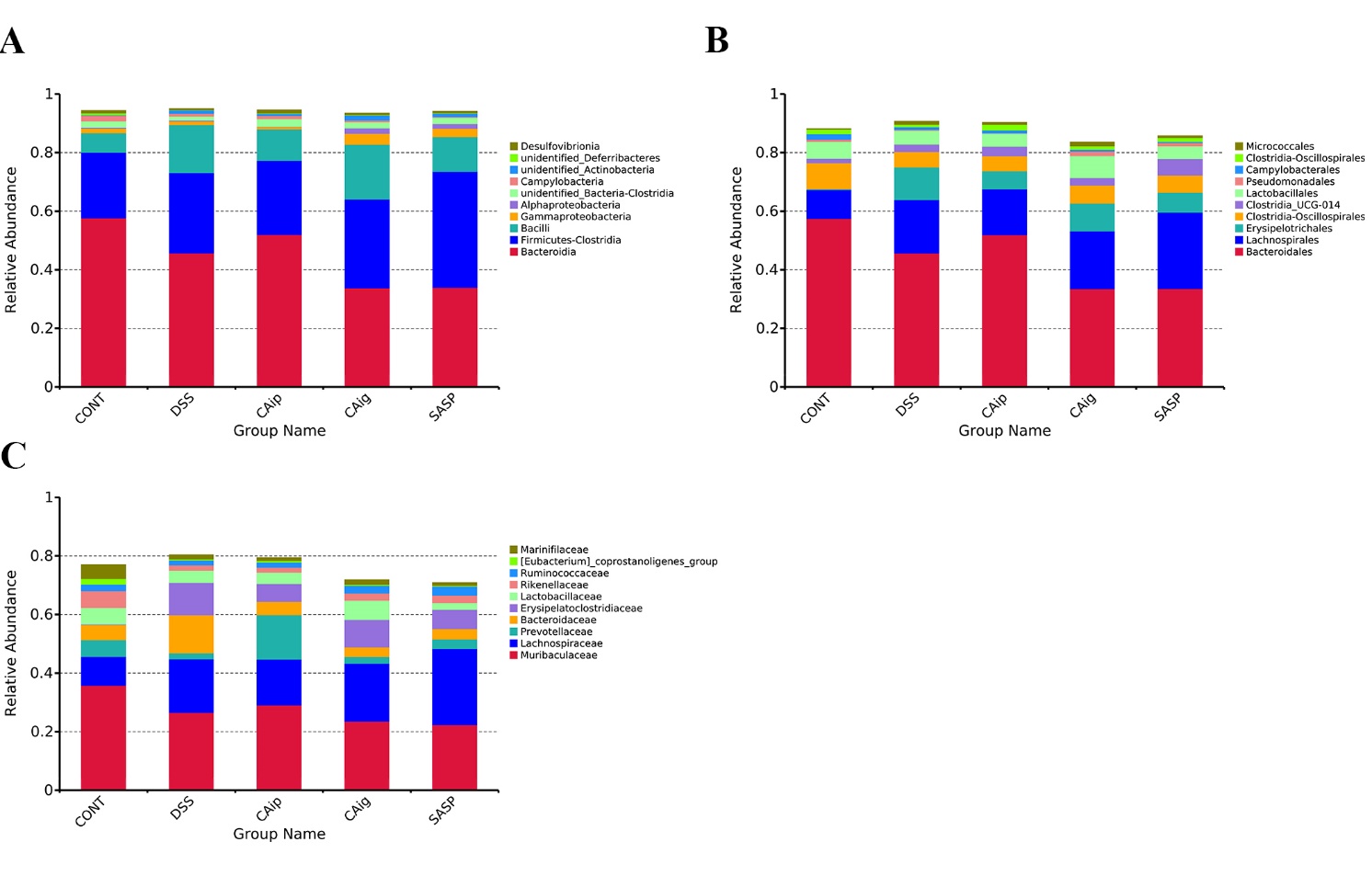


**Supplementary Figure 2.** The relative abundance of fecal microbiota in the top 10 of class (**A**), order (**B**) and family (**C**).

**
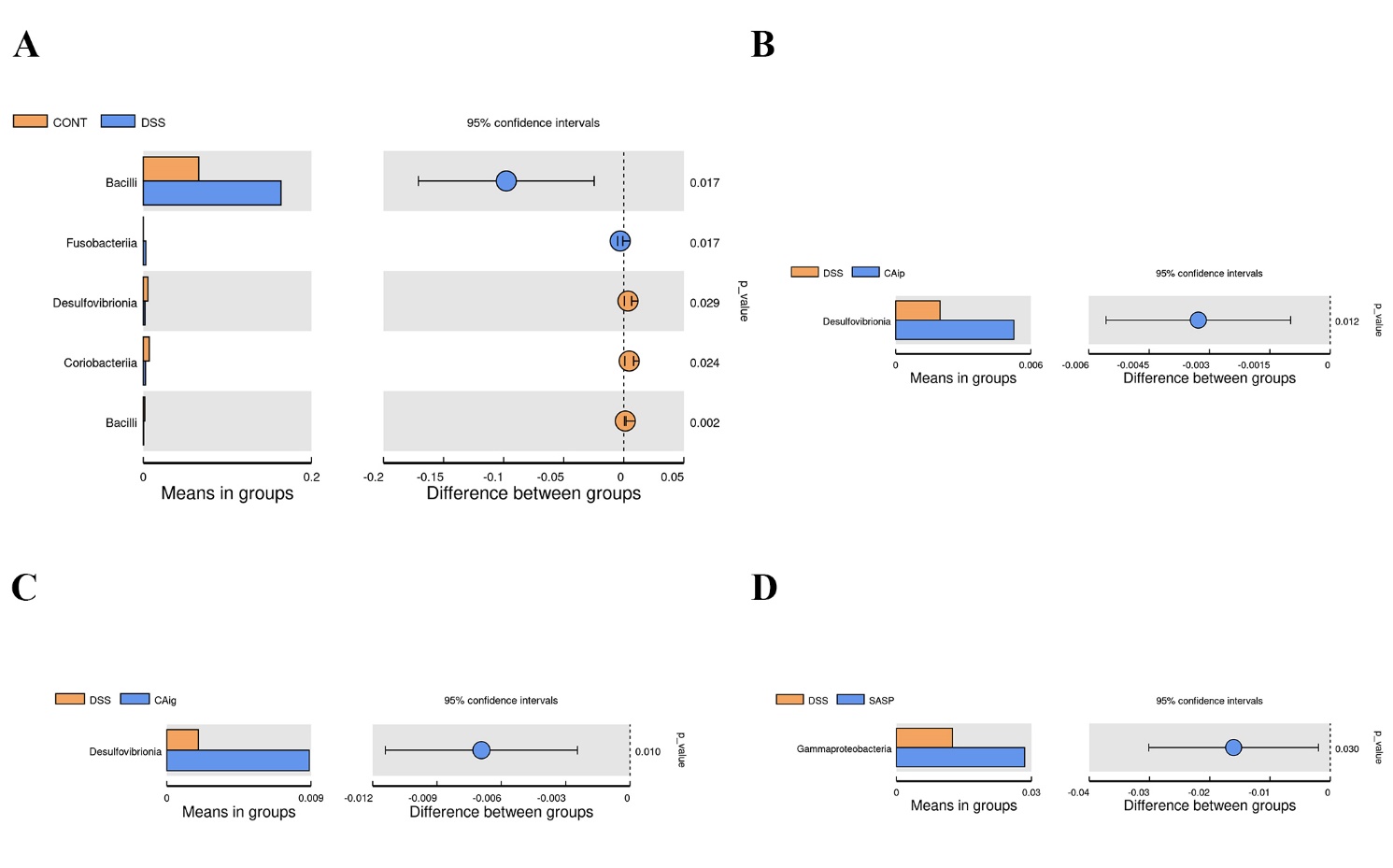
**

**Supplementary Figure 3.** Analysis of different species between groups at class level. (**A**) Control *vs* DSS; (**B**) DSS *vs* CAip; (**C**) DSS *vs* CAig; (**D**) DSS *vs* SASP. Statistical significance was evaluated by the Student’s T-test, *P* < 0.05.

**
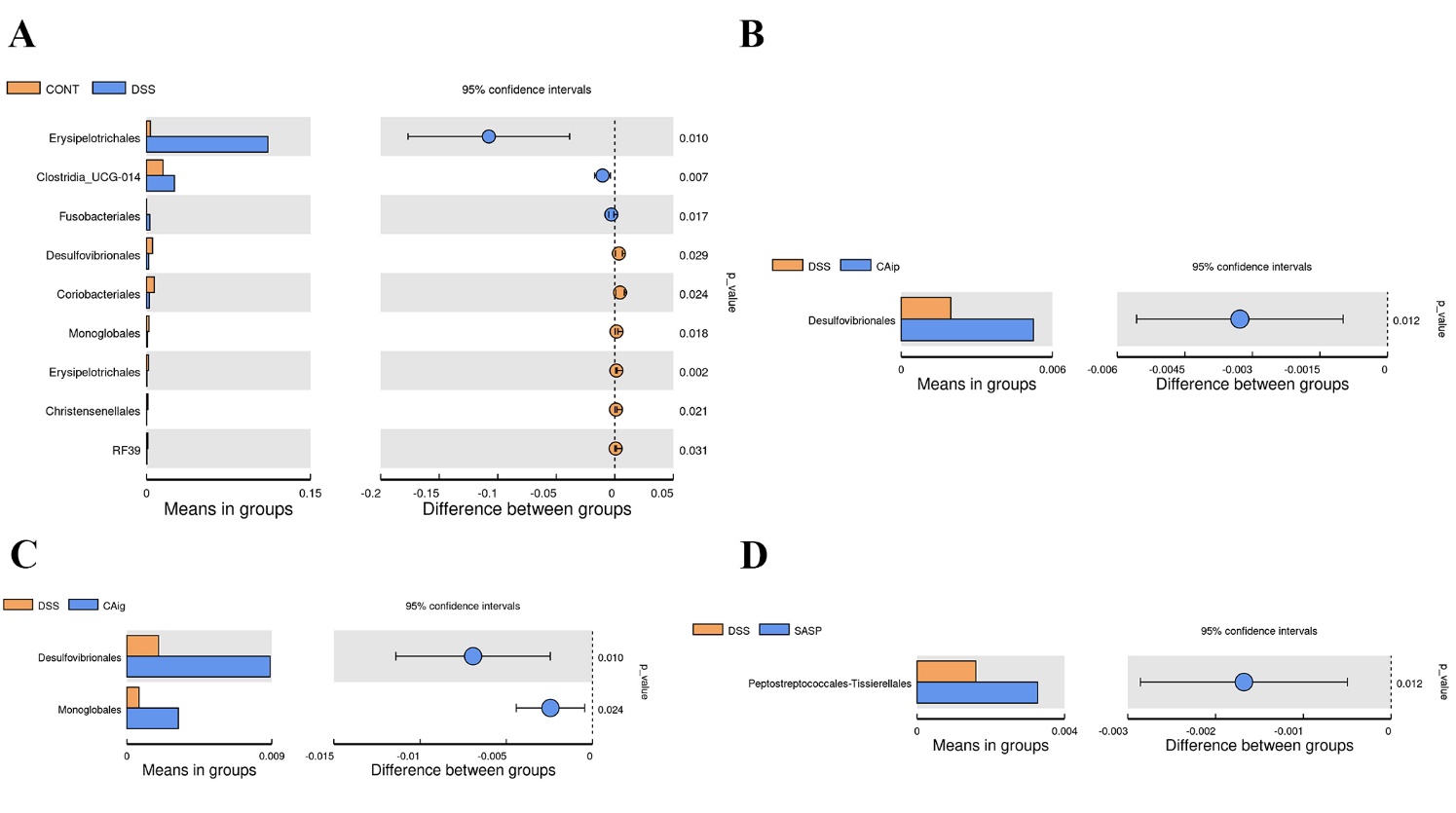
**

**Supplementary Figure 4.** Analysis of different species between groups at order level. (**A**) Control *vs* DSS; (**B**) DSS *vs* CAip; (**C**) DSS *vs* CAig; (**D**) DSS *vs* SASP. Statistical significance was evaluated by the Student’s T-test, *P* < 0.05.

**
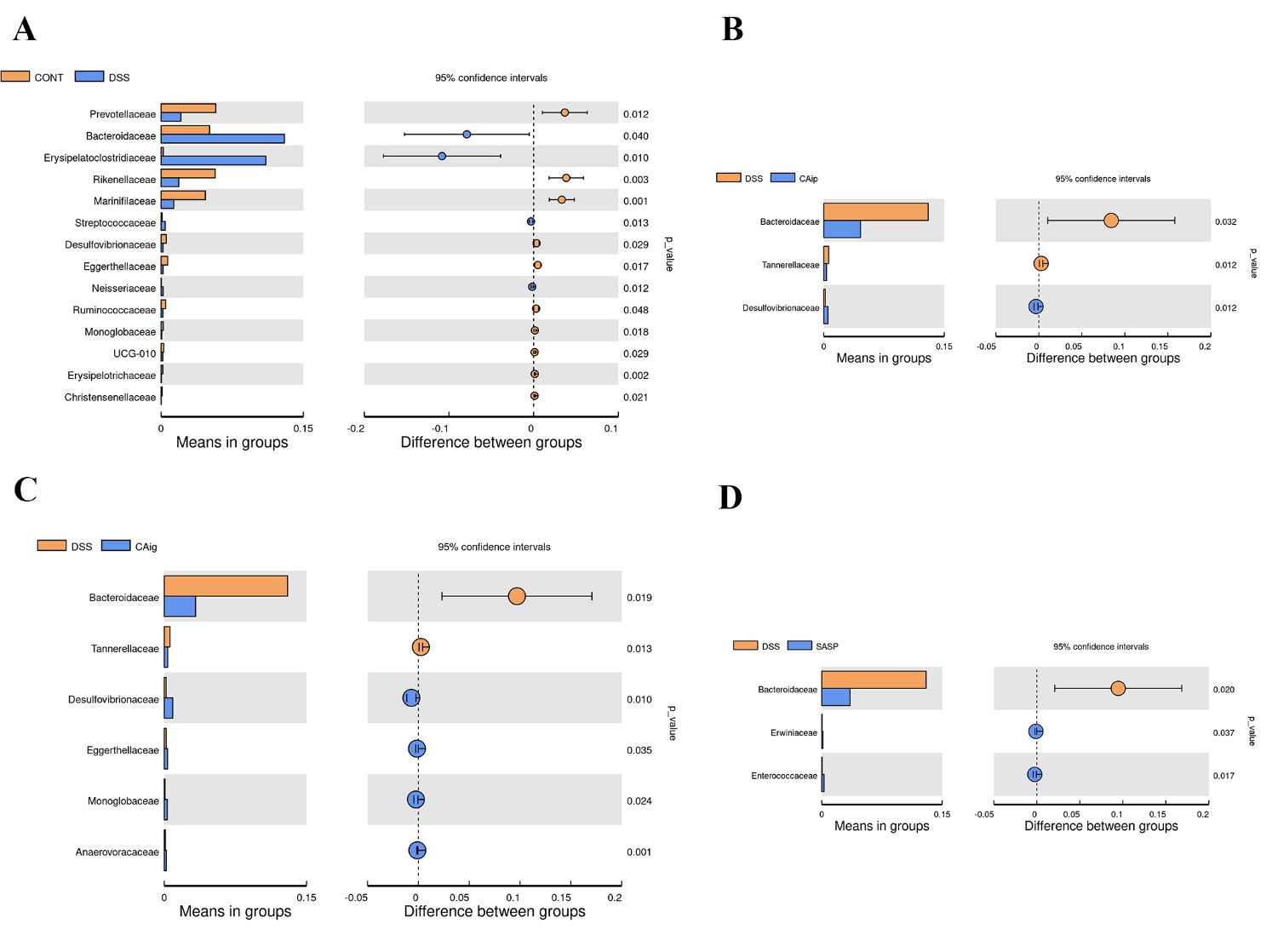
**

**Supplementary Figure 5.** Analysis of different species between groups at family level. (**A**) Control *vs* DSS; (**B**) DSS *vs* CAip; (**C**) DSS *vs* CAig; (**D**) DSS *vs* SASP. Statistical significance was evaluated by the Student’s T-test, *P* < 0.05.


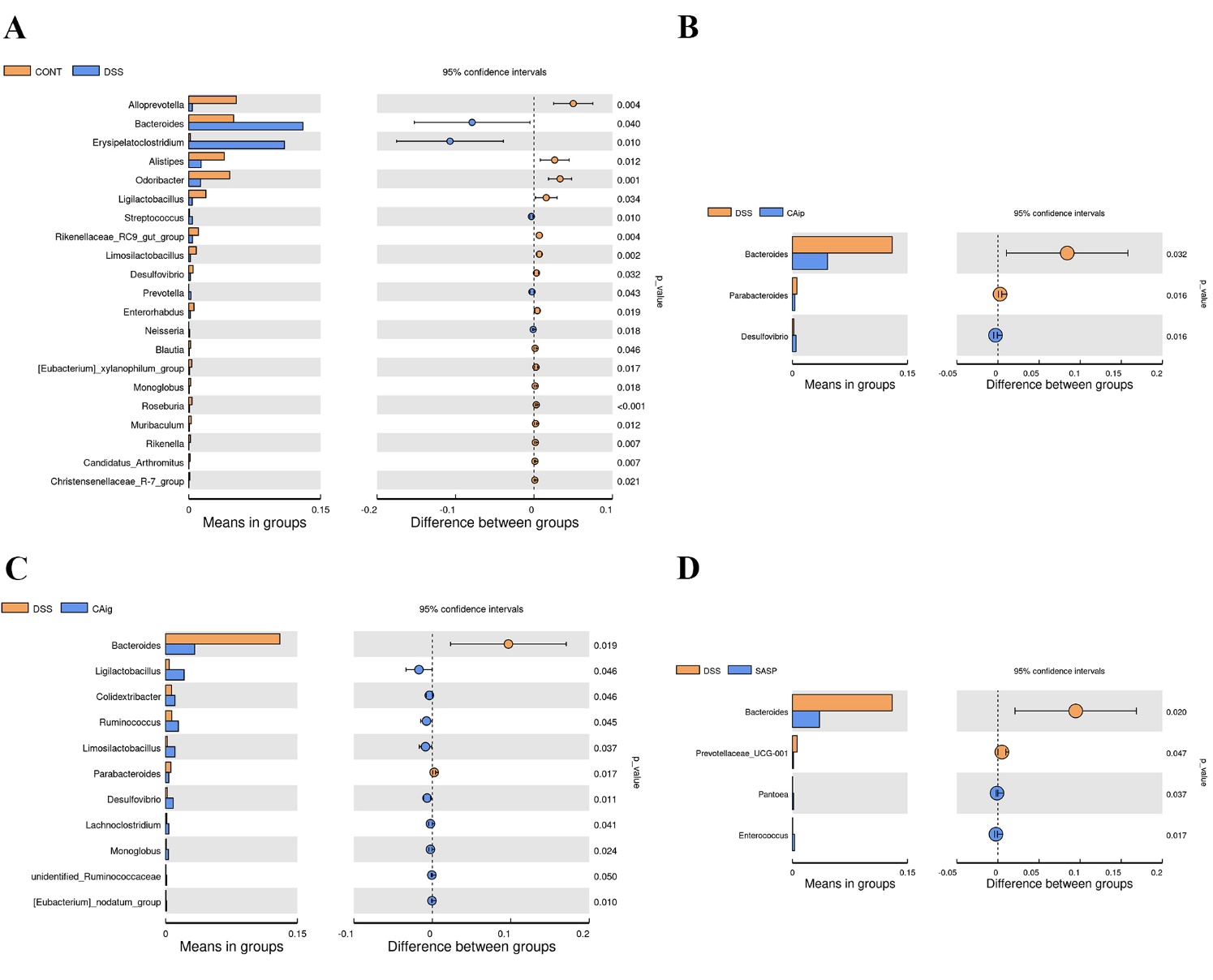


**Supplementary Figure 6.** Analysis of different species between groups at genus level. (**A**) Control *vs* DSS; (**B**) DSS *vs* CAip; (**C**) DSS *vs* CAig; (**D**) DSS *vs* SASP. Statistical significance was evaluated by the Student’s T-test, *P* < 0.05.


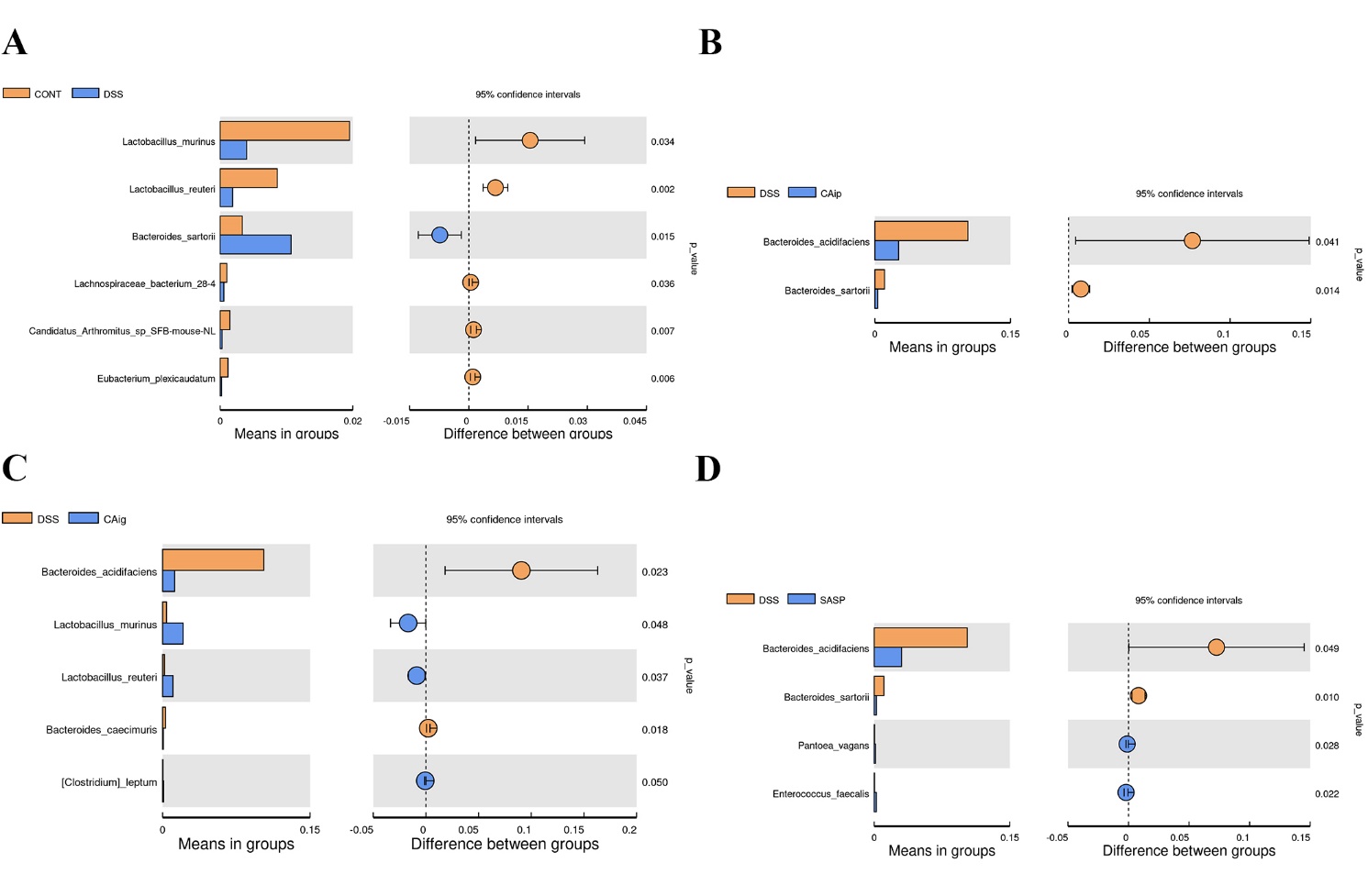


**Supplementary Figure 7.** Analysis of different species between groups at species level. (**A**) Control *vs* DSS; (**B**) DSS *vs* CAip; (**C**) DSS *vs* CAig; (**D**) DSS *vs* SASP. Statistical significance was evaluated by the Student’s T-test, *P* < 0.05.


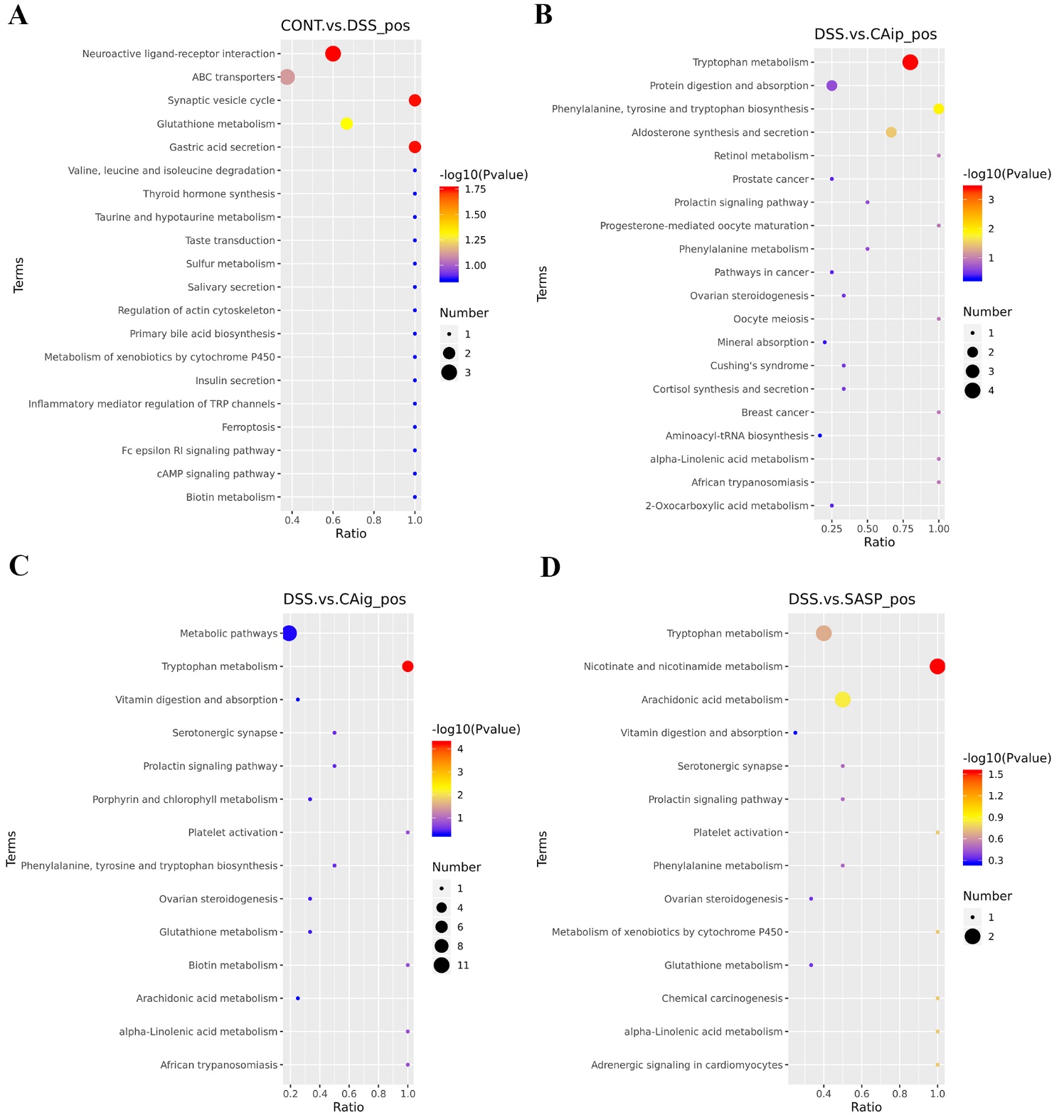


**Supplementary Figure 8.** KEGG metabolic pathway enrichment analysis in the positive mode. (**A**) Control *vs* DSS; (**B**) DSS *vs* CAip; (**C**) DSS *vs* CAig; (**D**) DSS *vs* SASP.


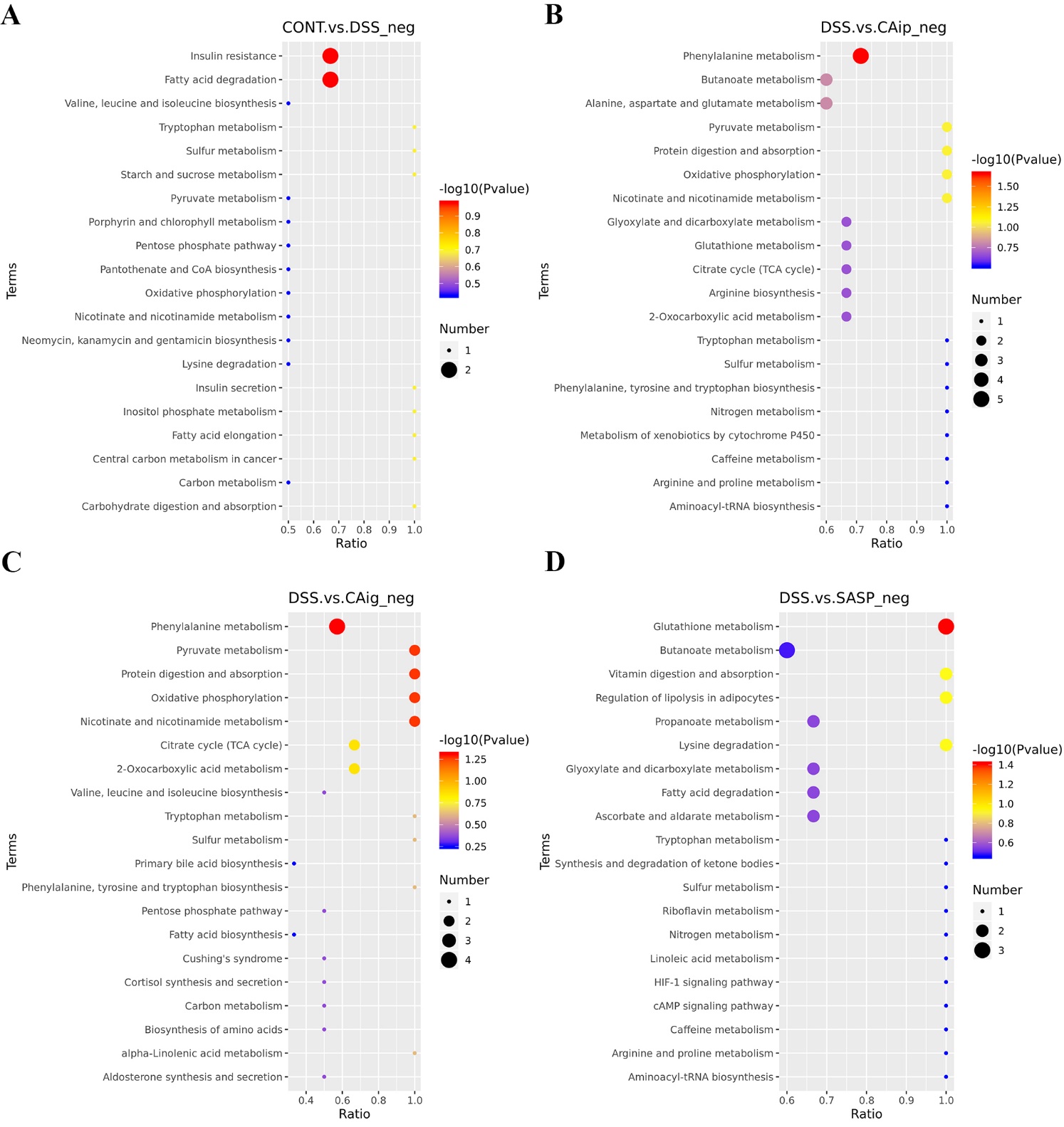


**Supplementary Figure 8.** KEGG metabolic pathway enrichment analysis in the negative mode. (**A**) Control *vs* DSS; (**B**) DSS *vs* CAip; (**C**) DSS *vs* CAig; (**D**) DSS *vs* SASP.

## Supplementary Table

**Supplementary Table 1.** Related gene and primer information.

| Gene | Orientation | Primer Sequences |
| --- | --- | --- |
| IL-6 | Forward | CTCCCAACAGACCTGTCTATAC |
|  | Reverse | CCATTGCACAACTCTTTTCTCA |
| IL-1β | Forward | CACTACAGGCTCCGAGATGAACAAC |
|  | Reverse | TGTCGTTGCTTGGTTCTCCTTGTAC |
| TNF-α | Forward | ATGTCTCAGCCTCTTCTCATTC |
|  | Reverse | GCTTGTCACTCGAATTTTGAGA |

**Supplementary Table 2.** Identification of significantly differential metabolites in the serum of Control and DSS-induced colitis mice with CA or SASP treatments.

| Metabolites | Formula | CONT&DSS | DSS&CAip | DSS&CAig | DSS&SASP | Pathway or explanation |
| --- | --- | --- | --- | --- | --- | --- |
| PC (16:0/19:2) | C_43_H_82_NO_8_P | ###(↓) | ***(↑) | *(↑) | **(↑) | Phosphatidylcholine |
| Mupirocin | C_26_H_44_O_9_ | ###(↑) | *(↓) | **(↓) | *(↓) | Medium-chain fatty acid |
| Oleamide | C_18_H_35_NO | ##(↓) | ***(↑) | **(↑) | **(↑) | Fatty amide |
| Kynurenic acid | C_10_H_7_NO_3_ | ##(↑) | ***(↓) | **(↓) | *(↓) | Tryptophan metabolism |
| 2-Amino-1,3,4-octadecanetriol | C_18_H_39_NO_3_ | ##(↓) | ***(↑) | ***(↑) | **(↑) | Phospholipid, a major component of all biological membrane |
| DL-Malic acid | C_4_H_6_O_5_ | ##(↑) | **(↓) | ***(↓) | *(↓) | Citrate cycle (TCA cycle) |
| Aspartylphenylalanine | C_13_H_16_N_2_O_5_ | ##(↑) | *(↓) | **(↓) | ***(↓) | Dipeptide |
| Succinic acid | C_4_H_6_O_4_ | #(↑) | ***(↓) | **(↓) | *(↓) | Oxidative phosphorylation; Pyruvate metabolism; Nicotinate and nicotinamide metabolism; TCA cycle |
| Methylsuccinic acid | C_5_H_8_O_4_ | ###(↑) | ***(↓) | *(↓) |  | Isoleucine catabolism |
| Eicosapentaenoic acid ethyl ester | C_22_H_34_O_2_ | ###(↓) | ***(↑) | *(↑) |  | Fatty acid ester |
| Phenylalanylproline | C_14_H_18_N_2_O_3_ | ###(↑) | **(↓) | ***(↓) |  | Dipeptide |
| Propionylcarnitine | C_10_H_19_NO_4_ | ###(↑) | **(↓) | *(↓) | **(↓) | Short-chain acylcarnitine |
| o-Tyrosine | C_9_H_11_NO_3_ | ##(↓) | ***(↑) | ***(↑) |  | Tyrosine metabolism |
| Oleoylethanolamide | C_20_H_39_NO_2_ | ##(↓) | ***(↑) | *(↑) |  | N-Acylethanolamine, constituent of the membrane-bound phospholipid |
| L-Kynurenine | C_10_H_12_N_2_O_3_ | ##(↑) | **(↓) | **(↓) |  | Tryptophan metabolism |
| N6-Succinyl Adenosine | C_14_H_17_N_5_O_8_ | ##(↑) | **(↓) | **(↓) |  | Purine nucleoside |
| 5-Methoxyindole-3-carbaldehyde | C_10_H_9_NO_2_ | ##(↑) | **(↓) | *(↓) |  | Indole derivative |
| N-lactoylphenylalanine | C_12_H_15_NO_4_ | #(↑) | **(↓) | ***(↓) |  | Lactoyl derivative of phenylalanine |
| D-Gluconic acid | C_6_H_12_O_7_ | #(↓) | *(↑) | *(↑) |  | Pentose phosphate pathway; Carbon metabolism |
| Glutaric Acid | C_5_H_8_O_4_ | ##(↑) | ***(↓) |  | ***(↓) | Fatty acid degradation; Lysine degradation |
| Pyroglutamic acid | C_5_H_7_NO_3_ | ###(↑) |  | **(↓) | ***(↓) | Glutathione metabolism |
| LysoPS 22:6 | C_28_H_44_NO_9_P | ##(↑) |  | **(↓) | *(↓) | Lysophosphatidylserine |
| LPE 18:3 | C_23_H_42_NO_7_P | #(↓) |  | ***(↑) | ***(↑) | Lysophosphatidylethanolamine |
| Alanyltryptophan | C_14_H_17_N_3_O_3_ | #(↑) |  | **(↓) | *(↓) | Dipeptide |
| PC (19:0/20:5) | C_47_H_84_NO_8_P | ##(↓) | ***(↑) |  |  | Phosphatidylcholine |
| PC (16:1e/22:4) | C_46_H_84_NO_7_P | ##(↓) | **(↑) |  |  | Phosphatidylcholine |
| 1-Stearoylglycerol | C_21_H_42_O_4_ | ##(↑) | *(↓) |  |  | Monoacylglyceride |
| PC (17:0/17:1) | C_42_H_82_NO_8_P | #(↓) | ***(↑) |  |  | Phosphatidylcholine |
| PC (22:6e/15:1) | C_45_H_78_NO_7_P | #(↓) | ***(↑) |  |  | Phosphatidylcholine |
| PC (18:1e/2:0) | C_28_H_56_NO_7_P | #(↓) | ***(↑) |  |  | Phosphatidylcholine |
| SM (d18:1/22:0) | C_45_H_91_N_2_O_6_P | #(↓) | **(↑) |  |  | Sphingomyelin |
| Gluconolactone | C_6_H_10_O_6_ | #(↑) | **(↓) |  |  | Lactone of D-gluconic acid |
| SM (d18:1/15:0) | C_38_H_77_N_2_O_6_P | #(↓) | *(↑) |  |  | Sphingomyelin |
| PC (18:1/18:2) | C_44_H_82_NO_8_P | #(↓) | *(↑) |  |  | Phosphatidylcholine |
| Biotin | C_10_H_16_N_2_O_3_S | ###(↓) |  | ***(↑) |  | Biotin metabolism; Vitamin digestion and absorption |
| 3-Hydroxybutyric acid | C_4_H_8_O_3_ | ##(↓) |  | ***(↑) |  | Ketone body |
| Chenodeoxycholic Acid | C_24_H_40_O_4_ | ##(↑) |  | *(↓) |  | Primary bile acid biosynthesis; Bile secretion; Cholesterol metabolism |
| Kojic acid | C_6_H_6_O_4_ | #(↑) |  | **(↓) |  | Pyranone derivative |
| PC (19:1/19:2) | C_46_H_86_NO_8_P | #(↓) |  | **(↑) |  | Phosphatidylcholine |
| L-Glutamic acid monosodium salt | C_5_H_8_NNaO_4_ | #(↑) |  | **(↓) |  | Monosodium glutamate |
| 7-Ketocholesterol | C_27_H_44_O_2_ | #(↓) |  | **(↑) |  | Oxidation product of cholesterol |
| gamma-Glutamylleucine | C_11_H_20_N_2_O_5_ | #(↓) |  | *(↑) |  | Dipeptide |
| Salicylic acid | C_7_H_6_O_3_ | ##(↑) |  |  | ***(↓) | Phenylalanine metabolism; Bile secretion |
| LPE 20:3 | C_25_H_46_NO_7_P | #(↓) |  |  | *(↑) | Lysophosphatidylethanolamine |
| Octadecanamine | C_18_H_39_N | #(↑) |  |  | *(↓) | monoalkylamine |
| Acetylcholine | C_7_H_15_NO_2_ | ###(↑) |  |  |  | Neuroactive ligand-receptor interaction; Synaptic vesicle cycle; Gastric acid secretion; cAMP signaling pathway |
| Biliverdin | C_33_H_34_N_4_O_6_ | ###(↓) |  |  |  | Porphyrin and chlorophyll metabolism |
| LPC 17:0 | C_25_H_52_NO_7_P | ###(↑) |  |  |  | Lysophosphatidylcholine |
| LPC 17:1 | C_25_H_50_NO_7_P | ##(↑) |  |  |  | Lysophosphatidylcholine |
| gamma-Glutamyltyrosine | C_14_H_18_N_2_O_6_ | ##(↓) |  |  |  | Dipeptide |
| L-Valine | C_5_H_11_NO_2_ | ##(↓) |  |  |  | ABC transporters; Valine, leucine and isoleucine degradation |
| 3-Methyl-2-oxobutanoic acid | C_5_H_8_O_3_ | ##(↓) |  |  |  | Valine, leucine and isoleucine biosynthesis and degradation |
| D-ribose 5-phosphate | C_5_H_11_O_8_P | ##(↑) |  |  |  | Pentose phosphate pathway |
| Metanephrine | C_10_H_15_NO_3_ | ##(↓) |  |  |  | Metabolite of epinephrine |
| 16-Hydroxyhexadecanoic acid | C_16_H_32_O_3_ | ##(↓) |  |  |  | Hydroxy fatty acid |
| Palmitic acid | C_16_H_32_O_2_ | ##(↓) |  |  |  | Fatty acid metabolism |
| Taurine | C_2_H_7_NO_3_S | #(↑) |  |  |  | Neuroactive ligand-receptor interaction; Taurine and hypotaurine metabolism |
| PC (18:0/18:0) | C_44_H_88_NO_8_P | #(↓) |  |  |  | Phosphatidylcholine |
| LPC 20:2 | C_28_H_54_NO_7_P | #(↑) |  |  |  | Lysophosphatidylcholine |
| LPC 19:1 | C_27_H_54_NO_7_P | #(↑) |  |  |  | Lysophosphatidylcholine |
| LPC 19:0 | C_27_H_56_NO_7_P | #(↑) |  |  |  | Lysophosphatidylcholine |
| LPC 22:0 | C_30_H_62_NO_7_P | #(↑) |  |  |  | Lysophosphatidylcholine |
| LPC 20:1 | C_28_H_56_NO_7_P | #(↑) |  |  |  | Lysophosphatidylcholine |
| Porphobilinogen | C_10_H_14_N_2_O_4_ | #(↑) |  |  |  | Under certain conditions, porphobilinogen can act as a phototoxin, a neurotoxin, and a metabotoxin |
| Lauric acid ethyl ester | C_14_H_28_O_2_ | #(↓) |  |  |  | Fatty acid ester |
| 8,15-Dihete | C_20_H_32_O_4_ | #(↑) |  |  |  | Eicosanoid |
| N-Acetylglycine | C_4_H_7_NO_3_ | #(↓) |  |  |  | N-acetyl amino acid |
| N-Acetyl-L-tyrosine | C_11_H_13_NO_4_ | #(↓) |  |  |  | N-acetyl amino acid |
| D-Glucose 6-phosphate | C_6_H_13_O_9_P | #(↑) |  |  |  | Insulin resistance; Carbohydrate digestion and absorption |
| Histamine | C_5_H_9_N_3_ | #(↑) |  |  |  | Neuroactive ligand-receptor interaction; Histidine metabolism |
| Tetrahydrocortisone | C_21_H_32_O_5_ | #(↑) |  |  |  | Steroid |
| Glutamylglutamine | C_10_H_17_N_3_O_6_ | #(↑) |  |  |  | Dipeptide |
| Allolithocholic acid | C_24_H_40_O_3_ | #(↑) |  |  |  | Bile acid, high concentration in patients with colon cancer |
| Indole-3-lactic acid | C_11_H_11_NO_3_ | #(↓) | **(↓) | *(↓) | *(↓) | Tryptophan metabolism |
| 2-Ketohexanoic acid | C_6_H_10_O_3_ | #(↓) | *(↓) | *(↓) |  | Medium-chain keto acid |
| Pregnenolone | C_21_H_32_O_2_ | #(↓) | *(↓) | *(↓) |  | Aldosterone synthesis and secretion; Cortisol synthesis and secretion |
| 2-Hydroxy-2-methylbutanoic acid | C_5_H_10_O_3_ | #(↓) | *(↓) |  |  | Hydroxy fatty acid |
| 3-Succinoylpyridine | C_9_H_9_NO_3_ | #(↓) |  |  | **(↓) | Nicotinate and nicotinamide metabolism |
| Boldione | C_19_H_24_O_2_ | #(↑) |  |  | *(↑) | Precursor to the anabolic steroid boldenone |
| Oxidized glutathione | C_20_H_32_N_6_O_12_S_2_ | #(↑) |  |  |  | Glutathione dimer, involved in the metabolomic disorder called leukotriene C4 synthesis deficiency pathway |
| L-arginine | C_6_H_14_N_4_O_2_ |  | ***(↑) | ***(↑) | ***(↑) | Amino acid |
| Indole-3-pyruvic acid | C_11_H_9_NO_3_ |  | ***(↓) | ***(↓) | *(↓) | Tryptophan metabolism |
| N-Formylkynurenine | C_11_H_12_N_2_O_4_ |  | ***(↓) | **(↓) | **(↓) | Tryptophan metabolism |
| Palmitic amide | C_16_H_33_NO |  | ***(↑) | *(↑) | *(↑) | Fatty acid amide |
| 5-Hydroxytryptophan | C_11_H_12_N_2_O_3_ |  | **(↓) | ***(↓) | *(↓) | Tryptophan metabolism |
| 6-Methylquinoline | C_10_H_9_N |  | **(↓) | ***(↓) | *(↓) | Quinoline derivatives |
| Citraconic acid | C_5_H_6_O_4_ |  | **(↓) | **(↓) | **(↓) | Valine, leucine and isoleucine biosynthesis |
| Jasmonic acid | C_12_H_18_O_3_ |  | **(↓) | **(↓) | *(↓) | Alpha-Linolenic acid metabolism |
| PC (18:1/19:2) | C_45_H_84_NO_8_P |  | *(↑) | **(↑) | *(↑) | Phosphatidylcholine |
| N5-Acetylornithine | C_7_H_14_N_2_O_3_ |  | *(↑) | **(↑) | *(↑) | Amino acid |
| Thromboxane B2 | C_20_H_34_O_6_ |  | *(↑) | **(↑) | *(↑) | Arachidonic acid metabolism |
| Glycyl-phenylalanine | C_11_H_14_N_2_O_3_ |  | *(↓) | **(↓) | *(↓) | Dipeptide |
| N-Acetyl-L-methionine | C_7_H_13_NO_3_S |  | ***(↓) | ***(↓) |  | N-acetylamino acid |
| Orotic acid | C_5_H_4_N_2_O_4_ |  | ***(↑) | *(↑) |  | Pyrimidine metabolism |
| Indole | C_8_H_7_N |  | **(↓) | ***(↓) |  | Tryptophan metabolism; Phenylalanine, tyrosine and tryptophan biosynthesis |
| Indole-3-acrylic acid | C_11_H_9_NO_2_ |  | **(↓) | ***(↓) |  | Tryptophan metabolism |
| 4-Hydroxy-3-methylbenzoic acid | C_8_H_8_O_3_ |  | **(↓) | **(↓) |  | Hydroxybenzoic acid |
| Phenylpyruvic acid | C_9_H_8_O_3_ |  | **(↓) | **(↓) |  | Phenylalanine metabolism |
| N-Acetyl-L-phenylalanine | C_11_H_13_O_3_ |  | **(↓) | *(↓) |  | Phenylalanine metabolism; Many N-acetylamino acid including N-acetylphenylalanine, are classified as uremic toxin |
| 4-Methylphenol | C_7_H_8_O |  | **(↓) | *(↓) |  | Metabolite of aromatic acid metabolism produced by intestinal microbiota in humans and animals;  A fecal biomarker of *clostridium difficile* infection |
| 2-Isopropylmalic acid | C_7_H_12_O_5_ |  | **(↓) | *(↓) |  | Hydroxy fatty acid |
| Indoleacetic acid | C_10_H_9_NO_2_ |  | **(↓) | *(↓) |  | Tryptophan metabolism |
| Fumaric acid | C_4_H_4_O_4_ |  | **(↓) | *(↓) |  | Oxidative phosphorylation; Pyruvate metabolism; Nicotinate and nicotinamide metabolism |
| Xanthurenic Acid | C_10_H_7_NO_4_ |  | *(↓) | **(↓) |  | Tryptophan metabolism |
| Purine | C_5_H_4_N_4_ |  | *(↓) | *(↓) |  | Purine metabolism |
| PC (14:0e/3:0) | C_25_H_52_NO_7_P |  | *(↑) | *(↑) |  | Phosphatidylcholine |
| Allantoin | C_4_H_6_N_4_O_3_ |  | *(↑) | *(↑) |  | Purine metabolism |
| Octadecanamide | C_18_H_37_NO |  | *(↑) | *(↑) |  | Fatty amide |
| Dehydroascorbic acid | C_6_H_6_O_6_ |  | ***(↓) |  | *(↓) | Oxidized form of ascorbic acid, may be a unique *E. coli* metabolite |
| 4-Hydroxy-2-oxoglutaric Acid | C_5_H_6_O_6_ |  | ***(↓) |  | *(↓) | Involved in the metabolic disorder called hyperornithinemia with gyrate atrophy |
| L-Glutamic acid | C_5_H_9_NO_4_ |  | **(↑) |  | *(↑) | Arginine and proline metabolism; Histidine metabolism; Taurine and hypotaurine metabolism |
| Capryloylglycine | C_10_H_19_NO_3_ |  | **(↓) |  | **(↓) | An acylglycine, normally minor metabolites of fatty acids, can be used to diagnose disorders associated with mitochondrial fatty acid beta-oxidation in certain cases |
| Glutaconic acid | C_5_H_6_O_4_ |  | **(↓) |  | *(↓) | Dicarboxylic acid |
| Palmitoylethanolamide | C_18_H_37_NO_2_ |  | *(↑) |  | **(↓) | N-Palmitoylethanolamide, present in the tissues of most mammals |
| PC (19:1/20:5) | C_47_H_82_NO_8_P |  | *(↑) |  | **(↑) | Phosphatidylcholine |
| Taurodeoxycholic acid | C_26_H_45_NNaO_6_S |  | *(↓) |  | **(↓) | Bile salt |
| Xanthosine | C_10_H_12_N_4_O_6_ |  | *(↑) |  | *(↑) | Purine metabolism |
| LPE 18:2 | C_23_H_44_NO_7_P |  |  | ***(↑) | **(↑) | Lysophosphatidylethanolamine |
| Prostaglandin G2 | C_20_H_32_O_6_ |  |  | ***(↓) | ***(↓) | Platelet activation; Arachidonic acid metabolism |
| 3-Hydroxyanthranilic acid | C_7_H_7_NO_3_ |  |  | **(↓) | *(↓) | Tryptophan metabolism |
| Prolylhydroxyproline | C_10_H_16_N_2_O_4_ |  |  | **(↓) | **(↓) | Dipeptide |
| cis-5,8,11,14,17-Eicosapentaenoic acid | C_20_H_30_O_2_ |  |  | **(↓) | *(↓) | An important polyunsaturated fatty acid, serves as the precursor for the prostanglandin-3 and thromboxane-3 families |
| LPC 14:1 | C_22_H_44_NO_7_P |  |  | *(↑) | ***(↓) | Lysophosphatidylcholine |
| LPS 20:4 | C_26_H_44_NO_9_P |  | ***(↑) |  |  | Lysophosphatidylserine |
| LPE 20:1 | C_25_H_50_NO_7_P |  | ***(↑) |  |  | Lysophosphatidylethanolamine |
| Stearoylethanolamide | C_20_H_41_NO_2_ |  | ***(↑) |  |  | N-acetylethanolamine (NAE), NAEs constitute a class of lipid compounds naturally present in both animal and plant membranes as constituents of the membrane-bound phospholipid |
| Glycerol 3-phosphate | C_3_H_9_O_6_P |  | ***(↑) |  |  | Glycolysis metabolic pathway |
| L-Phenylalanine | C_9_H_11_NO_2_ |  | **(↓) |  |  | Amino acid |
| 4-Oxoretinol | C_20_H_28_O_2_ |  | **(↑) |  |  | Metabolite of retinol |
| Thromboxane B3 | C_20_H_32_O_6_ |  | *(↑) |  |  | Prostanoid |
| Octanedioic acid | C_8_H_14_O_4_ |  | *(↓) |  |  | Dicarboxylic acid, is found to be associated with carnitine-acylcarnitine translocase deficiency |
| PC (16:0e/2:0) | C_26_H_54_NO_7_P |  | *(↑) |  |  | Phosphatidylcholine |
| PC (20:3/20:3) | C_48_H_84_NO_8_P |  | *(↑) |  |  | Phosphatidylcholine |
| PC (18:2e/2:0) | C_28_H_54_NO_7_P |  | *(↑) |  |  | Phosphatidylcholine |
| 10-Nitrolinoleic acid | C_18_H_31_NO_4_ |  | *(↓) |  |  | Lineolic acid derivative |
| Deoxycorticosterone | C_21_H_30_O_3_ |  | *(↓) |  |  | Steroid hormone |
| 2-Methylpentanedioic acid | C_6_H_10_O_~~4~~_ |  | *(↓) |  |  | Methyl-branched fatty acid |
| Arachidic acid | C_20_H_40_O_2_ |  | *(↓) |  |  | Long-chain fatty acid |
| Progesterone | C_21_H_30_O_2_ |  | *(↓) |  |  | Aldosterone synthesis and secretion; Cortisol synthesis and secretion |
| Vitamin E acetate | C_31_H_52_O_3_ |  | *(↑) |  |  |  |
| Bilirubin | C_33_H_36_N_4_O_6_ |  |  | **(↓) |  | Porphyrin and chlorophyll metabolism; Bile secretion |
| β-Muricholic acid | C_24_H_40_O_5_ |  |  | *(↑) |  | Bile acid |
| Stearic acid | C_18_H_36_O_2_ |  |  | *(↑) |  | Long-chain fatty acid, C18:0 |
| Monobutylphthalate | C_12_H_14_O_4_ |  |  | *(↑) |  | Benzoic acid ester |
| (S)-beta-Aminoisobutyric acid | C_4_H_9_NO_2_ |  |  | *(↓) |  | Valine metabolism |
| SM (d21:0/15:1) | C_41_H_83_N_2_O_6_P |  |  | *(↑) |  | Sphingomyelin |
| SM (d15:1/23:0) | C_43_H_87_N_2_O_6_P |  |  | *(↑) |  | Sphingomyelin |
| PC (19:2/19:2) | C_46_H_84_NO_8_P |  |  | *(↑) |  | Phosphatidylcholine |
| Methyltestosterone | C_20_H_30_O_2_ |  |  | *(↓) |  | Steroid |
| 7-Ketolithocholic acid | C_24_H_38_O_4_ |  |  | *(↑) |  | Bile acid |
| Cis-4-Hydroxyproline | C_5_H_9_NO_3_ |  |  | *(↓) |  | Proline derivative |
| Paracetamol | C_8_H_9_NO_2_ |  |  |  | ***(↑) | Acetaminophen |
| Palmitoylcarnitine | C_23_H_45_NO_4_ |  |  |  | ***(↓) | Long-chain acylcarnitine |
| Octadeca-11E,13E,15Z-trienoic acid | C_18_H_30_O_2_ |  |  |  | **(↓) | Fatty acid |
| Phenylacetylglycine | C_10_H_11_NO_3_ |  |  |  | **(↓) | Acyl glycine, a putative biomarker of phospholipidosis |
| L-Ascorbate | C_6_H_8_O_6_ |  |  |  | **(↓) | Vitamin C |
| N-Tigloylglycine | C_7_H_11_NO_3_ |  |  |  | **(↓) | Acyl glycine |
| 10-Undecenoic acid | C_11_H_20_O_2_ |  |  |  | **(↓) | Unsaturated fatty acid |
| Pentadecanoic acid | C_15_H_30_O_2_ |  |  |  | **(↓) | Straight chain fatty acid |
| Lauric acid | C_12_H_24_O_2_ |  |  |  | **(↓) | Fatty acid biosynthesis |
| Estrone | C_18_H_22_O_2_ |  |  |  | **(↓) | Steroid |
| N-Acetyl-L-leucine | C_8_H_15_NO_3_ |  |  |  | **(↓) | N-acetylamino acid |
| Hippuric acid | C_9_H_9_NO_3_ |  |  |  | **(↑) | Acyl glycine |
| Tetrahydrocorticosterone | C_21_H_34_O_4_ |  |  |  | **(↓) | Steroid |
| 5,6-dihydroxyindole-2-carboxylic acid | C_9_H_7_NO_4_ |  |  |  | **(↑) | Indolecarboxylic acid derivative |
| Decanoylcarnitine | C_17_H_33_NO_4_ |  |  |  | **(↓) | Medium-chain acylcarnitine |
| 12-Hydroxydodecanoic acid | C_12_H_24_O_3_ |  |  |  | *(↓) | Medium-chain hydroxy acid |
| LPE 17:0 | C_22_H_46_NO_7_P |  |  |  | *(↑) | Lysophosphatidylethanolamine |
| LPE 19:0 | C_24_H_50_NO_7_P |  |  |  | *(↑) | Lysophosphatidylethanolamine |
| Tetradecanedioic acid | C_14_H_26_O_4_ |  |  |  | *(↓) | Fatty Acid |
| Hexanoylcarnitine | C_13_H_25_NO_4_ |  |  |  | *(↓) | Medium-chain acylcarnitine |
| 5-OxoETE | C_20_H_30_O_3_ |  |  |  | *(↓) | Long-chain fatty acid |
| Nicotinamide | C_6_H_6_N_2_O |  |  |  | *(↑) | Nicotinamide metabolism |
| Palmitoleic acid | C_16_H_30_O_2_ |  |  |  | *(↓) | Omega-7 monounsaturated fatty acid |
| Cortisol | C_21_H_30_O_5_ |  |  |  | *(↓) | Glucocorticoid |
| Alanylalanine | C_6_H_12_N_2_O_3_ |  |  |  | *(↑) | Dipeptide |
| 13,14-dihydro-15-keto-PGD2 | C_20_H_32_O_5_ |  |  |  | *(↑) | Metabolite of PGD2 |
| N4-Acetylcytidine | C_11_H_15_N_3_O_6_ |  |  |  | *(↓) | Endogenous urinary nucleoside, biological markers for patients with colorectal cancer |
| Taurodeoxycholic acid | C_26_H_44_NNaO_6_S |  |  |  | *(↓) | A bile salt formed in the liver by conjunction of deoxycholate with taurine |
| Orotidine | C_10_H_12_N_2_O_8_ |  |  |  | *(↓) | Intermediate in the biosynthesis of pyrimidine nucleotide |
| 4-Acetamidobutyric acid | C_6_H_11_NO_3_ |  |  |  | *(↓) | Gamma amino acid derivative |
| L-Adrenaline | C_9_H_13_NO_3_ |  |  |  | *(↑) | Has suppressive effect on the immune system. |
| 2-Methylbutyroylcarnitine | C_12_H_23_NO_4_ |  |  |  | *(↓) | Short-chain acylcarnitine |
| LPE 16:0 | C_21_H_44_NO_7_P |  |  |  | *(↑) | Lysophosphatidylethanolamine |
| LPC 20:4 | C_28_H_50_NO_7_P |  |  |  | *(↓) | Lysophosphatidylcholine |
| Arachidonic acid | C_20_H_32_O_2_ |  |  |  | *(↓) | Essential fatty acid, a constituent of animal phosphatides |
| Gentisuric acid | C_7_H_6_O_4_ |  |  |  | *(↑) | A metabolite of aspirin |

“↑” indicates increase, “↓” indicates decrease

“#” indicates significance difference of DSS *vs* Control (^###^ *P* < 0.001, ^##^ *P* < 0.01, ^#^ *P* < 0.05); “*” indicates significant difference of treatment groups *vs* DSS (^***^ *P* < 0.001, ^**^ *P* < 0.01, ^*^ *P* < 0.05).
